# Structured Conducting Polymers Templated from Ouzo-Induced Oxidant Superstructures for High-Performance Supercapacitors

**DOI:** 10.3390/polym18182260

**Published:** 2026-09-16

**Authors:** Doo-Ho Kang, Jia Lee, Dahl-Young Khang

**Affiliations:** Department of Materials Science and Engineering, Yonsei University, Seoul 03722, Republic of Korea; doohoya12@yonsei.ac.kr (D.-H.K.); jia501@naver.com (J.L.)

**Keywords:** chemical oxidative polymerization, conducting polymers, Ouzo emulsion, oxidant superstructures, supercapacitors

## Abstract

Conducting polymers (CPs) that are structured in very specific shapes have been successfully synthesized via vapor phase chemical oxidative polymerization of monomers with oxidant superstructures as templates. A ternary mixture of iron(III) p-toluenesulfonate hexahydrate (Fe(pts)_3_·6H_2_O) as the oxidant, n-butanol as the solvent, and cyclohexane as the anti-solvent has been found to form an unstable Ouzo emulsion, leading to the precipitation of the oxidant within emulsion drops into nano-sized particles. Interestingly, the formed oxidant nanoparticles then aggregated spontaneously into specific shapes, forming superstructures. The oxidant superstructures took the shape of micron-scale rods initially, which underwent shape transformation into a few micron-sized plates, and then finally into tens of microns-long nanofibers. These oxidant superstructures (rods, plates, or fibers) have been found to retain their oxidizing properties for the polymerization of various CPs, enabling us to prepare structured CPs in rod, plate, and fiber shapes. Especially, the fiber-shaped oxidant superstructures were used for the synthesis of nanofibrous PEDOT films for supercapacitors. The PEDOT nanofiber film exhibited a specific capacitance of 114.3 F/g, significantly higher than that of the dense PEDOT film (54.2 F/g), with capacitance retention of 73% under rate testing thanks to its electrochemical stability. Furthermore, the PEDOT nanofiber film exhibited lower charge transfer resistance (R_ct_) and equivalent series resistance (ESR) compared to the dense film, demonstrating superior charge transfer characteristics at the interface.

## 1. Introduction

Conducting polymers (CPs) have long been developed for diverse applications. For the chemical synthesis of CPs such as polyethylenedioxythiophene (PEDOT), polypyrrole (Ppy), polythiophene (PT), etc., oxidizing agents for each monomer are essential, regardless of solution or vapor phase synthesis [1,2,3,4,5,6]. Typical oxidants that have been widely used in the chemical oxidative polymerization of CPs include hydrated iron-based salts such as iron(III) p-toluenesulfonate hexahydrate (Fe(pts)_3_·6H_2_O) and iron(III) chloride hexahydrate (FeCl_3_·6H_2_O) [4,7,8], among others. These oxidants are applied as either solution or solid film forms for the polymerization, leading to CP dispersion or a solid film, respectively.

To synthesize the CPs in a predefined shape, one can employ templates, including hard templates of metals, metal oxides, carbon structures, and polymers [9,10,11] or soft templates that are composed of self-assembled structures of micelles, foams, or emulsions [9,10,12,13]. The most common hard-template method involves using particles such as silica, polymer beads, carbon, metals, or metal oxides as templates to form the structures. While this approach allows for the creation of well-defined structures, the coating and subsequent removal of the template can be complex and may risk damaging the structure. Alternatively, soft-template and self-template methods are frequently employed. The soft-template method utilizes self-assembly phenomena involving micelles, vesicles, foams, or emulsions, whereas the self-template method relies on specific chemical reactions or etching rates determined by crystal facets to form nanostructures. In the case of the soft-template method, surfactants play a crucial role in forming nanostructures, but they may leave impurities that negatively affect the physical properties, such as electrical conductivity, of the final product.

Recently, we have demonstrated the surfactant-less stable Ouzo emulsion, as a template, for the synthesis of hollow nanospheres and collapsed bowls of CPs [14]. Contrary to the other template-based approaches mentioned above, Ouzo emulsion does not involve any foreign substances. In other words, Ouzo emulsion spontaneously forms when proper amounts of oxidant, solvent, and anti-solvent are mixed together, with no need to add any additional additives [15,16,17]. A similar but slightly different condition for Ouzo emulsion leads to the formation of an unstable Ouzo emulsion, yielding precipitated nanoparticles. Then, the formed nanoparticles aggregate via oriented attachment to form larger, few μm-scale objects, i.e., superstructures.

Superstructures are the assemblies of nanoparticles that, under certain conditions, appear as a single large crystal due to the ordered alignment of constituent nanoparticles [18,19,20,21,22]. Classical crystallization theory explains that monomers gradually add to form clusters, which grow into crystals [23,24]. However, recent studies have shown that non-classical pathways involving the aggregation of higher-order structures can also lead to superstructure formation [25,26,27,28,29]. In this process, oriented attachment occurs, creating various intermediate states that ultimately result in complex and hierarchical final structures. Such superstructures possess a high surface area and porous structure, which result in excellent electrical and chemical properties. They are particularly advantageous for their high catalytic efficiency and ability to form naturally without complex processes. Furthermore, superstructures exhibit high stability and electrochemical performance, making them suitable for use in energy storage devices, such as batteries and supercapacitors, as well as in catalysts, solar cells, light-emitting diodes (LEDs), drug delivery systems and biosensors [30,31,32,33].

In this work, we demonstrate the synthesis of structured CPs, such as PEDOT, Ppy and PTTh, using oxidant superstructures as templates. Similar to the case of the stable Ouzo emulsion shown previously, a ternary system, with Fe(pts)_3_·6H_2_O as an oxidant, butanol as a solvent, and cyclohexane as an anti-solvent, has led to the formation of different shapes of oxidant superstructures. Contrary to the formation of a stable Ouzo emulsion, a slightly different combination of oxidant/solvent/anti-solvent has resulted in an unstable Ouzo emulsion, leading to the precipitation of oxidant nanoparticles. Then, the formed nanoparticles aggregate to form oxidant superstructures. Interestingly, it has been found that the oxidant superstructures take the shape of rods, plates, and fibers, depending on the solution stirring time. The generation and following transformation in the shapes of oxidant superstructures have been found to depend on various parameters, such as the type of solvent and anti-solvent, and the existence of extra added water quantity.

With the chemical oxidative polymerization with monomers via either solution- or vapor-phase routes, using those oxidant superstructures as templates, structured CPs have been successfully prepared in rod, plate, or fiber shapes. After dissolving the oxidant template, the final polymers are in those shapes and hollow inside. Among them, hollow nanofibrous PEDOT film has been prepared and tested for electrodes in supercapacitors. Thanks to the void spaces inside PEDOT fibers as well as spaces among nanofibers, the supercapacitor has shown very high specific capacitance (114.3 F/g), in comparison to the capacitance (54.2 F/g) of PEDOT dense film. In addition, electrochemical impedance spectroscopy (EIS) has revealed that the hollow nanofiber sample has lower charge transfer resistance (R_ct_) and equivalent series resistance (ESR) compared to the dense film counterpart, indicating superior charge transfer characteristics at the electrode–electrolyte interface. The formation of oxidant superstructures and template-based chemical oxidative polymerization of hollow CP rods/plates/fibers can be quite useful in various fields, including energy storage devices.

## 2. Materials and Methods

Iron(III) p-toluenesulfonate hexahydrate ((CH_3_C_6_H_4_SO_3_)_3_Fe·6H_2_O), 1-butanol (ACS reagent, ≥99.4%), and 1-hexanol were purchased from Sigma-Aldrich (St. Louis, MO, USA). 3,4-ethylenedioxythiophene (EDOT, 97%), pyrrole (98+%), and 2,2′:5′,2″-terthiophene (99%) were purchased from Thermo Fisher Scientific (Waltham, MA, USA). Toluene, cyclohexane, ethyl alcohol and 2-propanol were purchased from Samchun (Pyeongtaek-si, Republic of Korea) and used as received.

An oxidant solution was prepared by dissolving 16.6 wt% of Fe(pts)_3_·6H_2_O (or iron tosylate) in 1-butanol and stirring for 1 h at room temperature until completely dissolved. Then, 200 µL of the oxidant solution was added to a large amount of anti-solvent, 50 mL of cyclohexane, and stirred at 400 rpm in a 30 °C water bath to maintain the temperature. As stirring continued, the yellowish solution gradually transformed into a pale-yellow suspension (Figure S1). When the stirring stopped, it was observed that the precipitated pale-yellow particles in the suspension settled down at the bottom of the clear solution. These particles are superstructures in the anti-solvent, and they transform in shape from rods, plates, and finally into nanofibers, in sequence, during stirring.

The prepared oxidant superstructures in anti-solvent were cast onto Si or glass substrates by spinning at 4000 rpm for 1 min in order to quickly dry the solvent and anti-solvent. Then, the coated substrates and 100 µL of monomer (EDOT and pyrrole) were placed in a preheated, closed petri dish at 40 °C for polymerization via the vapor phase polymerization (VPP) route. Different temperatures and reaction times were used to control the thickness and final structure. Meanwhile, for polyterthiophene (PTTh), since the melting point of the monomer is 95 °C, 0.01 g of the monomer powder was placed in a preheated, closed petri dish and heated at 100 °C until the reaction occurred. After the polymerization, the samples were rinsed with ethanol twice, each for 10 min, to remove the oxidant and then dried at room temperature.

The nanofiber-shaped oxidant superstructure solution was spray-coated onto ITO glass (10 Ω/□) in order to obtain a uniform and percolated network film. The ITO glass was cut into 1 cm × 1.5 cm pieces; cleaned with acetone, 2-propanol, and de-ionized (DI) water with sonication; and then dried in an oven. The cleaned ITO substrate was masked with polyimide (PI) tape, ensuring the coating was confined to an area of 1 cm × 1 cm. A spray gun with 0.35 MPa nitrogen as the carrier gas was used. The substrate was heated on a hotplate at ~38 °C during spray-coating to evaporate the solvent immediately. For PEDOT polymerization, the coated ITO substrate and 100 µL of EDOT droplet were placed in a preheated, sealed petri dish at 40 °C for VPP. After the reaction, the sample was rinsed with ethanol twice, each for 10 min, to remove the oxidant and then dried at room temperature.

The samples were investigated by SEM (S-5000, Hitachi, Tokyo, Japan) and transmission electron microscopy (TEM; JEM-F200, JEOL, Tokyo, Japan). X-ray diffraction (XRD) analysis was carried out using a Rigaku Smartlab (Tokyo, Japan) diffractometer operated at 45 kV and 200 mA with Cu-Kα radiation source (λ = 0.154 nm): scan step 0.02°, 20 mm × 20 mm window slit. And the scan range (2θ) was 3° to 80°. The chemical structures of Fe(pts)_3_·6H_2_O and synthesized PEDOT fiber film were analyzed by a Fourier-transform infrared spectrometer (FT-IR; Vertex 70, Bruker, Billerica, MA, USA) in the range of 650–4000 cm^−1^. X-ray photoelectron spectroscopy (XPS; K-Alpha, Thermo, London, UK) was performed to analyze the chemical composition of pristine and heated powder of Fe(pts)_3_·6H_2_O. The obtained XPS spectra were analyzed using CASA XPS software (version 2.3.18PR1.0) in which the background was simulated using a Shirley function and the peaks were fitted using a Gaussian–Lorentzian function. All the binding energies were referenced to C1s (284.6 eV) for calibration. The Raman spectra of PEDOT fiber film were measured using a Raman spectrometer (LabRam Aramis; Horiba Jobin Yvon, Palaiseau, France) with a Nd:YAG laser (532 nm). The absorption of PEDOT fiber film was measured using a UV–Vis spectrometer (V-670; JASCO, Tokyo, Japan) in the range of 300 to 2400 nm. The 4-point probe (4PP; CMT-SR2000N, Advanced Instrument Technologies, Inc., Norwood, MA, USA) was used to measure the sheet resistance of various samples, and the measured sheet resistance values were converted into electrical conductivities with separately measured (by cross-sectional SEM) sample thicknesses. Atomic force microscopy (AFM; MFP-3D, Asylum Research, Santa Barbara, CA, USA) was used to characterize the surface details of the polymerized PEDOT.

The electrochemical performance of PEDOT nanofiber-coated on ITO glass was measured using a three-electrode system through galvanostatic charge–discharge, cyclic voltammetry, and EIS with a potentiostat (Figure S2). The composition of the three-electrode system is as follows: a PEDOT fiber-coated ITO substrate was used as the working electrode (WE), a platinum wire served as the counter electrode (CE), and an Ag/AgCl electrode was used as the reference electrode (RE), with 1 M KCl aqueous solution as an electrolyte (at room temperature and normal pressure). The cyclic voltammetry (CV) and galvanostatic charge–discharge (GCD) tests were conducted within a voltage range of −0.2 V to 0.7 V, at varying rates, using a potentiostat (VMP-3e, Biologic, Seyssinet-Pariset, France). The CV scan rate ranged from 5 mV/s to 250 mV/s, while the GCD charge/discharge rate varied from 0.1 A/g to 2 A/g. For the EIS test, measurements were taken in the frequency range of 0.01 Hz to 200 kHz at an AC amplitude of 5 mV. The specific mass capacitances of the material were calculated using GCD, according to Equation (1):

(1) $$C_{s}=\frac{I\times \Delta t}{\Delta V}$$

Here, *I*, Δ*t*, and Δ*V* represent the current density applied to the sample (specific current, A/g), discharge time (s), and voltage band (V), respectively [34].

## 3. Results

### 3.1. Formation of Oxidant Superstructures

**Figure 1:**
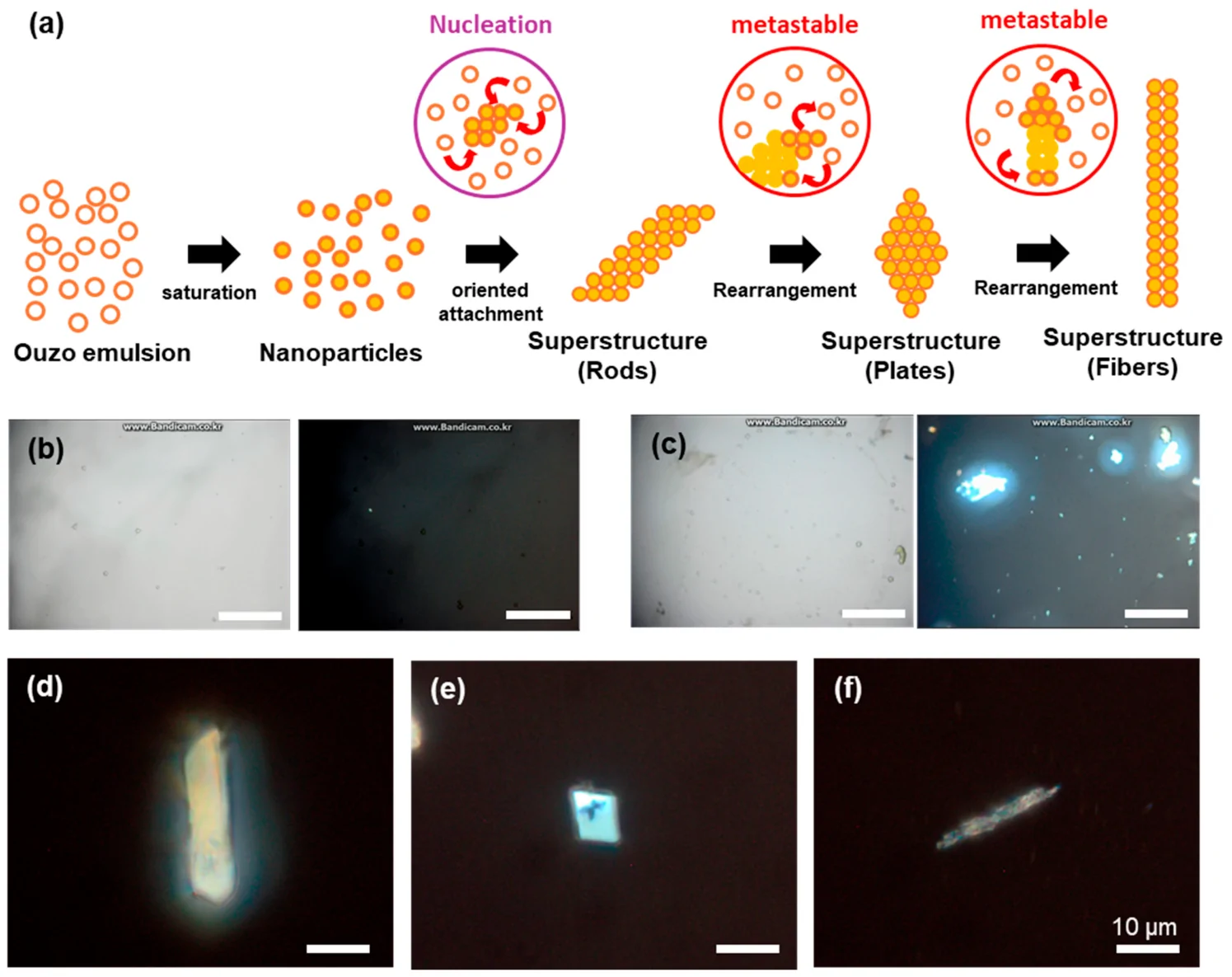
Oxidant superstructures. (**a**) Schematic illustration of steps involved in the superstructure formation. OM images of aliquot emulsion droplets at (**b**) 1 min and (**c**) 5 min after the ternary mixing. Images on the right in (**b**,**c**) were taken under polarized light to clearly visualize the crystallized particles. (**d**–**f**) OM images of solid superstructures in (**d**) rod, (**e**) plate, and (**f**) fiber shapes, respectively.

Figure 1a shows the steps involved in the formation of oxidant superstructures schematically. When the oxidant/butanol solution is added into the anti-solvent cyclohexane, the ternary mixture becomes cloudy with a yellow color, indicating the presence of droplets that scatter light or an Ouzo emulsion. Contrary to our previous report on the formation of a stable Ouzo emulsion [14], the present Ouzo emulsion is not stable; due to the mutual solubility between solvent (butanol) and anti-solvent (cyclohexane), the butanol in the emulsion droplets starts to dissolve into the bulk of the anti-solvent. In the meanwhile, the anti-solvent cyclohexane cannot penetrate into the emulsion droplets due to the mutual immiscibility between the oxidant and cyclohexane. The gradual drainage of butanol from the emulsion droplets leads to an increase in the oxidant concentration inside the droplets. Also, the concentration of the oxidant is much higher (~3.3 times) than that used for the stable Ouzo experiments [14]. Once the butanol is significantly depleted, the emulsion droplets reach a supersaturated state, leading to the precipitation of the oxidant. As shown in Figure 1b, the ternary mixture remains as emulsion droplets at ~1 min after the mixing. However, the droplets turn into shining objects after ~5 min of mixing, as shown in Figure 1c. In Figure 1b,c, the OM images with the dark background were taken under polarized light to clearly visualize the formed solid objects.

Interestingly, the precipitated nanoparticles subsequently coalesced into larger superstructures. The nanoparticle assembly is by an oriented attachment, resulting in a well-defined directional growth rather than random aggregation. Furthermore, the formed superstructures underwent three distinct morphological transformations based on stirring time after mixing. From the start of ternary mixing up to ~2 h, rod-shaped superstructures were predominant, as shown in Figure 1d. Then, upon further stirring, at ~3 h, most of the rod structures disappeared; instead, there appeared thin and diamond-shaped plate structures, as shown in Figure 1e. Upon further stirring of the ternary mixture, ~6 h or later, almost all the plates disappeared and there remained thin and long fibrous structures only in the solution, as shown in Figure 1f.

The ternary system used in this work is quite similar to our previous work [14] in that all three components are mutually miscible (or soluble), except the immiscibility between the oxidant and anti-solvent. In our previous work, the ternary system consisted of FeCl_3_·6H_2_O as an oxidant, acetone as a solvent, and toluene as an anti-solvent. This ternary system led to the formation of a stable Ouzo emulsion spontaneously, which has been used as a template for the synthesis of CP hollow nanostructures. On the other hand, the present work involves Fe(pts)_3_·6H_2_O as an oxidant, butanol as a solvent, and cyclohexane as an anti-solvent. This ternary system led to the formation of an unstable Ouzo emulsion, leading to the precipitation of nanoparticles and the formation of various superstructures. The drastic difference between the two systems, stable or unstable Ouzo emulsion formation, is in the larger (~3.3 times) concentration of oxidant in the present system. Under this high concentration of oxidant, the ternary mixture becomes unstable, i.e., outside of the stable Ouzo region in the ternary phase diagram shown in our previous work.

**Figure 2:**
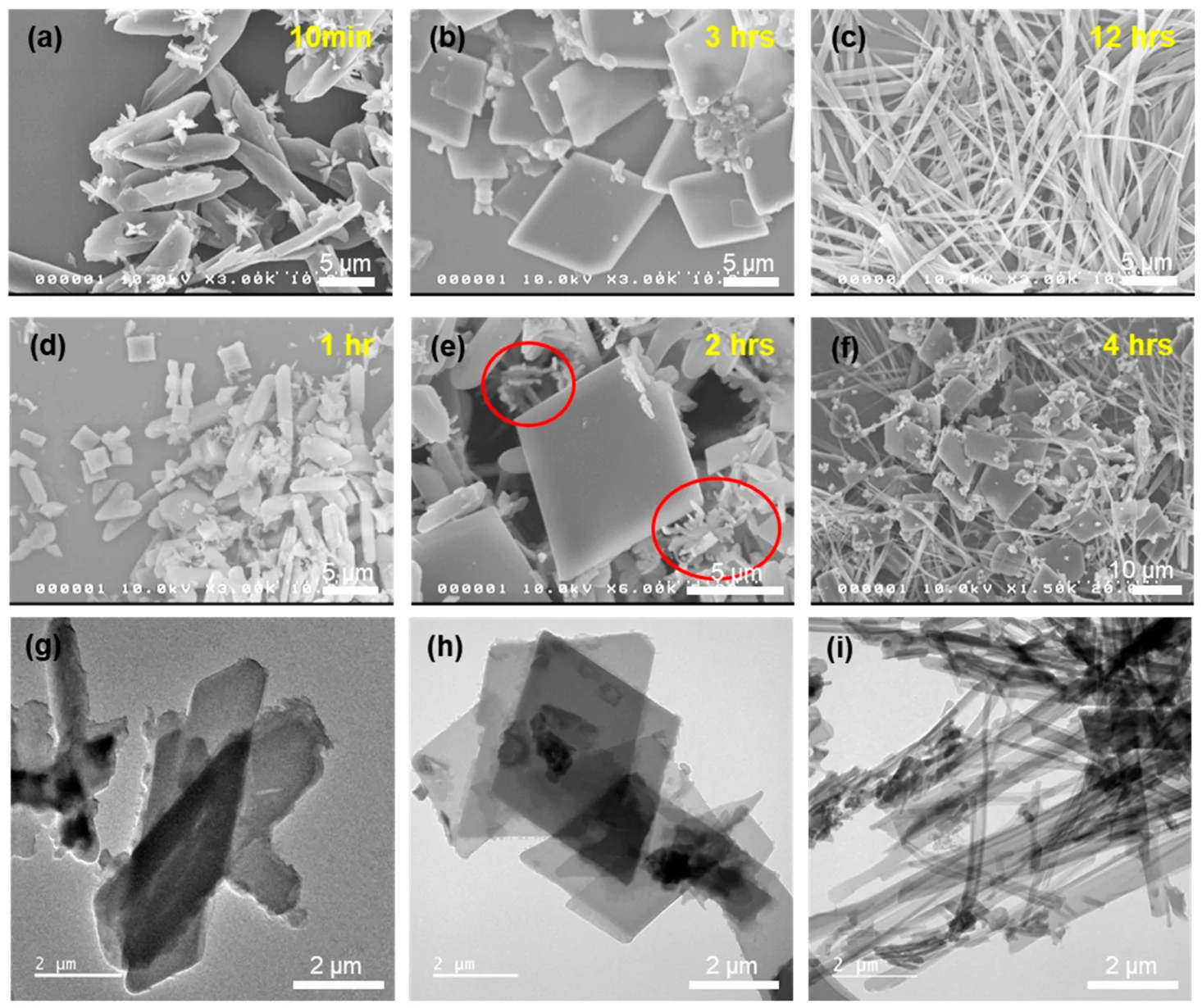
High-resolution images of oxidant superstructures. (**a**–**c**) SEM images of superstructures formed at different stages of stirring: rods at short time, plates later, and long nanofibers in the end, respectively. (**d**–**f**) Sample images in the middle of shape transformation. Small plate seeds are formed in (**d**), followed by plate formation at the expense of rods (**e**), and fibers are generated by dissolving the plates and rearranging into fibers (**f**). (**g**–**i**) TEM images of rods, plates and fibers, respectively.

To investigate further details of the formed various superstructures, higher resolution imaging techniques such as SEM and TEM were used, and the results are shown in Figure 2. The rod-shaped superstructures, in dimensions of ~10 μm long and 2–3 μm wide, were formed in the early stage of ternary mixing, as shown in Figure 2a for an aliquot after 10 min of stirring. Then, the rods were transformed into another superstructure of a thin plate shape at ~3 h of stirring, as shown in Figure 2b. The lateral dimension of the plate-shaped superstructure is ~10 μm × 10 μm. At the later stage of stirring, ~12 h, the plate-shaped superstructures totally disappeared, with the appearance of fiber-shaped ones, as shown in Figure 2c. The fiber-shaped superstructures are a few tens of micrometers long with a diameter of a few tens of nanometers. It should be noted that the observed shape transformation, from rod to plate and to fiber eventually, has occurred sequentially during the ternary mixture stirring, without any other control over the experimental conditions.

The intermediate states during the shape transformation were further elucidated by imaging samples near the borderlines of transformation. Figure 2d shows the sample imaged after 1 h of stirring, which is the state that the rod-shaped superstructures started to transform into plate-shaped ones. As shown, there appear small-sized plates, although the rod-shaped superstructures are still prevalent. At 2 h of stirring, shown in Figure 2e, there are large plate-shaped superstructures at the expense of rod-shaped ones. The encircled parts in red of Figure 2e show the reminiscence of rod-shaped superstructures, where the rods are mostly dissolved away to form plates and becoming a dendritic shape. Similarly, at the stirring time of 4 h, there appear fibrous objects (Figure 2f): upon further observation (Figure S3), the fiber-shaped superstructures seem to emanate from the surface of plate-shaped superstructures. All these observations clearly indicate that the shape transformation is a continuous process: samples taken at an arbitrary stirring time (within 6~8 h of stirring) contain a mixture of differently shaped superstructures, although there are prevalent superstructures depending on the specific stirring time. By stopping the stirring of the ternary mixture at certain moments, one can obtain the prevalent shape of superstructures, as shown in Figure 2a–c, which can be used as templates for the synthesis of structured CPs later on. Further, those superstructures were imaged by TEM: rods in Figure 2g, plates in Figure 2h, and fibers in Figure 2i.

It should be noted that the formation of various superstructures has been found in other ternary systems (Figure S4). With Fe(pts)_3_·6H_2_O as the oxidant and cyclohexane as the anti-solvent, other solvents such as isopropyl alcohol (IPA) and hexanol could also lead to superstructure formation, although there are subtle differences, such as the time for the occurrence of specific superstructures and the shapes of obtained superstructures. For example, in the oxidant/IPA/cyclohexane ternary system, rod-shaped superstructures have transformed directly into fiber-shaped ones, with no occurrence of intermediate plate-shaped superstructures. Further, toluene could be used as the anti-solvent, instead of cyclohexane, for the generation of various superstructures (Figure S4). With toluene as the anti-solvent, however, all the solvents used in this work (IPA, butanol, and hexanol) have led to the formation of rod- and fiber-shaped superstructures only, without any occurrence of plate-shaped superstructures, in addition to differences in the time at which each shape of superstructures starts to occur or disappear. These similar but subtle differences in superstructure formation, depending on the solvent/anti-solvent combinations, is likely due to the slight difference in mutual affinity or miscibility among various combinations. Nevertheless, by choosing a proper combination of oxidant/solvent/anti-solvent, one can obtain a desired shape of oxidant superstructures.

**Figure 3:**
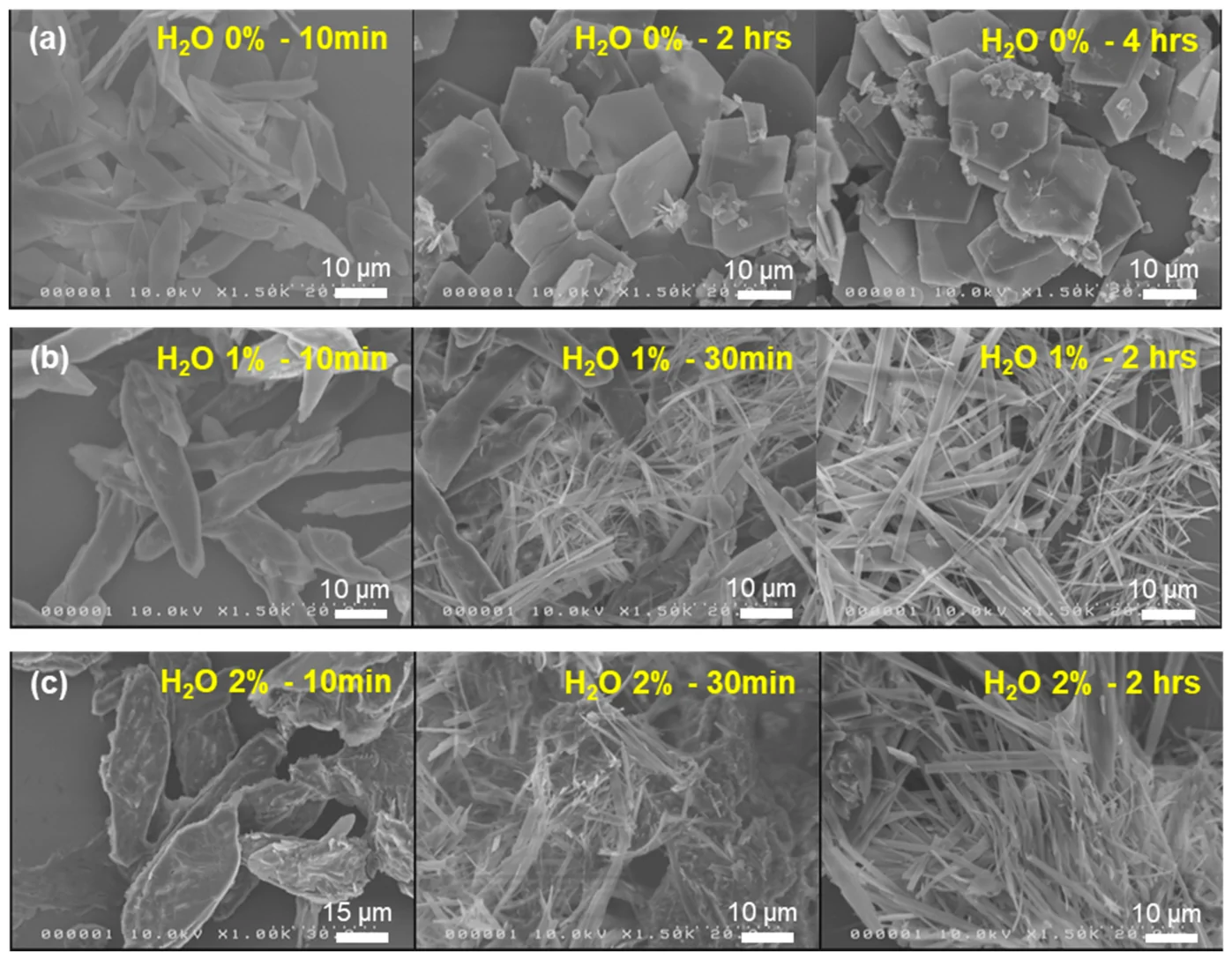
Effect of added water on superstructure formation. (**a**) Without any water addition, (**b**) 1 wt% water addition, and (**c**) 2 wt% water addition.

To obtain further insights on the mechanism of superstructure formation and their shape transformation, experiments have been performed by adding a small amount of water into the ternary system, as shown Figure 3. Without any added water, the superstructures take the shapes of rods, plates, and fibers, in sequence, upon continuous stirring. Shown in Figure 3a, rod-shaped superstructures transformed into plate shape in ~2 h, and the plate-shaped superstructures were maintained up to 4 h of stirring (upon further stirring, the plate-shaped superstructures transformed into fiber-shaped ones, as shown in Figure 2). With a small amount of added water, however, the shape of superstructures, their shape transformation, and the time of occurrence for a specific shape of superstructure were totally different compared to the case of without added water. Shown in Figure 3b are the images of superstructures formed with 1 wt% of added water. As shown, the formed rod-shaped superstructures have directly transformed into fiber-shaped ones in 30 min of stirring, skipping the formation of plate-shaped superstructures. Similarly, with 2 wt% of added water, rod-shaped superstructures have directly transformed into fiber-shaped ones. In addition, the surface of rod-shaped objects seems very rough, which means that the constituent nanoparticles were already partially dissolving to form fiber-shaped superstructures. In all, the formed shapes, shape transformation, and the time of occurrence of superstructures are critically affected by the added small amount of water. The added water molecule may coordinate Fe^3+^, expediting the formation of the most stable form of superstructure, oxidant fibers, and will be discussed below.

**Figure 4:**
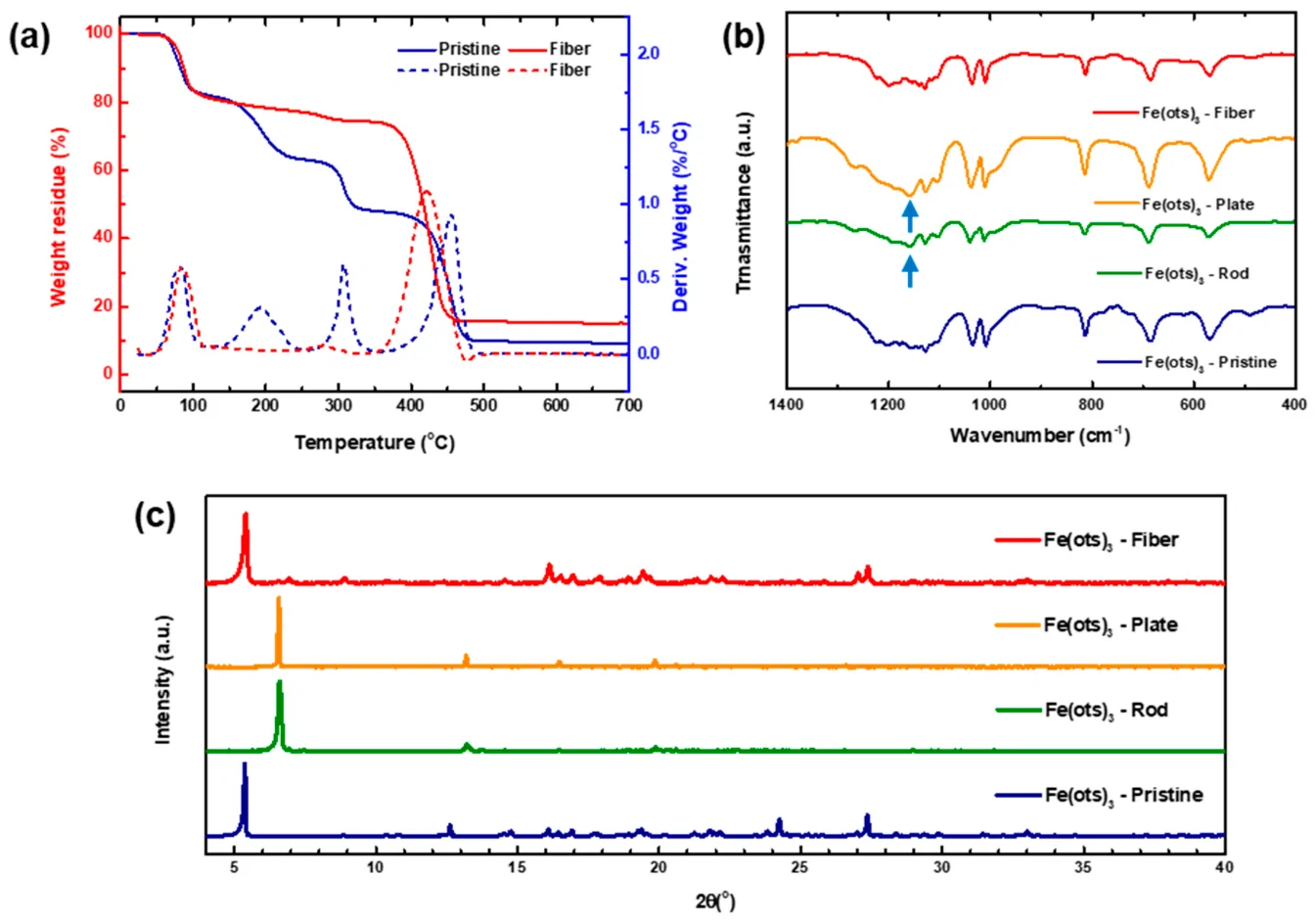
Analytic characterization of oxidant superstructures. (**a**) TGA scans, (**b**) FT-IR spectra, and (**c**) XRD spectra of pristine oxidant powder and different shapes of superstructures.

The formed superstructures have been characterized by various analytic techniques, and the results are shown in Figure 4. TGA data, shown in Figure 4a, show a drastic difference between pristine oxidant powders and other forms of oxidant superstructures (Figure S5). The pristine oxidant powders underwent multi-step decomposition: after dehydration at ~100 °C, becoming anhydrous Fe(pts)_3_ up to ~330 °C, then one tosylate group leaves becoming Fe(pts)_2_ upon further heating between ~330 °C and ~600 °C, and finally full decomposition into Fe_7_S_8_ and Fe_3_O_4_ after ~600 °C [35]. On the other hand, the superstructures of rod/plate/fiber show dehydration at ~100 °C, followed by full decomposition at ~600 °C. That is, the superstructures do not undergo stepwise decomposition. Instead, they maintain the dehydrated Fe(pts)_3_ over a large temperature span before their full decomposition at ~600 °C, which means the superstructures are objects formed via strong bonding between Fe^3+^ and tosylate anions.

In FT-IR spectra, all samples show typical absorption peaks for Fe(pts)_3_·6H_2_O reported in the literature [35,36]. The FT-IR spectra over the full wavenumber range can be found in Figure S6. A subtle difference among the samples can be found in the absorption peak at ~1145 cm^−1^ observed in the spectra for rod and plate superstructure samples, which is from symmetric stretching of SO_3_ and indicated by blue arrows in Figure 4b. This peak is one of the characteristic peaks for Fe(pts)_2_ [36]. That is, the rod- and plate-shaped superstructures show the FT-IR peak that is unique to Fe(pts)_2_, while the pristine powders and fiber-shaped oxidant superstructures do not have such a peak. Thus, the rod- and plate-shaped oxidant superstructures are likely to have insufficient bonding with tosylate anions, which makes them metastable and thus transforms them into stable ones, i.e., fiber-shaped superstructures.

Figure 4c shows the XRD spectra for pristine powders and various shapes of oxidant superstructures. A noticeable difference can be found in the strong peak around 2θ of 5~7°, which means that the superstructures have a crystalline order regardless of their specific shapes. While the pristine oxidant powders and fiber-shaped superstructures show the strong diffraction peak at ~5.3°, the rod- and plate-shaped counterparts show the diffraction peak shifted to the higher angle of ~6.5°. The inter-planar distance for pristine powders and fiber-shaped superstructures is calculated to be 1.67 nm, while that for rod- and plate-shaped superstructures is 1.36 nm. Considering the sequential shape transformation from rod, plate, to fiber in the end, the intermediate metastable rod- and plate-shaped superstructures have a more compact crystalline order due to a smaller inter-planar distance compared to the pristine oxidant powders and the final fiber-shaped superstructures. Combined with FT-IR data that show the characteristic peak of Fe(pts)_2_ in rod- and plate-shaped superstructures, the rod and plate superstructures have incomplete bonding between Fe^3+^ and tosylate anions, leading to a smaller inter-planar spacing in the XRD spectra. On the other hand, fiber superstructures seem to have enough bonding with tosylate, based on the FT-IR spectrum, and thus a slightly enlarged inter-planar distance in XRD. In all, rod and plate superstructures seem to be not in a stable state, and therefore, they are transformed into a stable form of fiber-shaped superstructures in the end. The added water expedites the formation of fibers (Figure 3), which seems that the water molecules may enhance the linkage between Fe and tosylate via H-bonding with the sulphonate group in tosylate.

### 3.2. Synthesis of Structured Conducting Polymers

**Figure 5:**
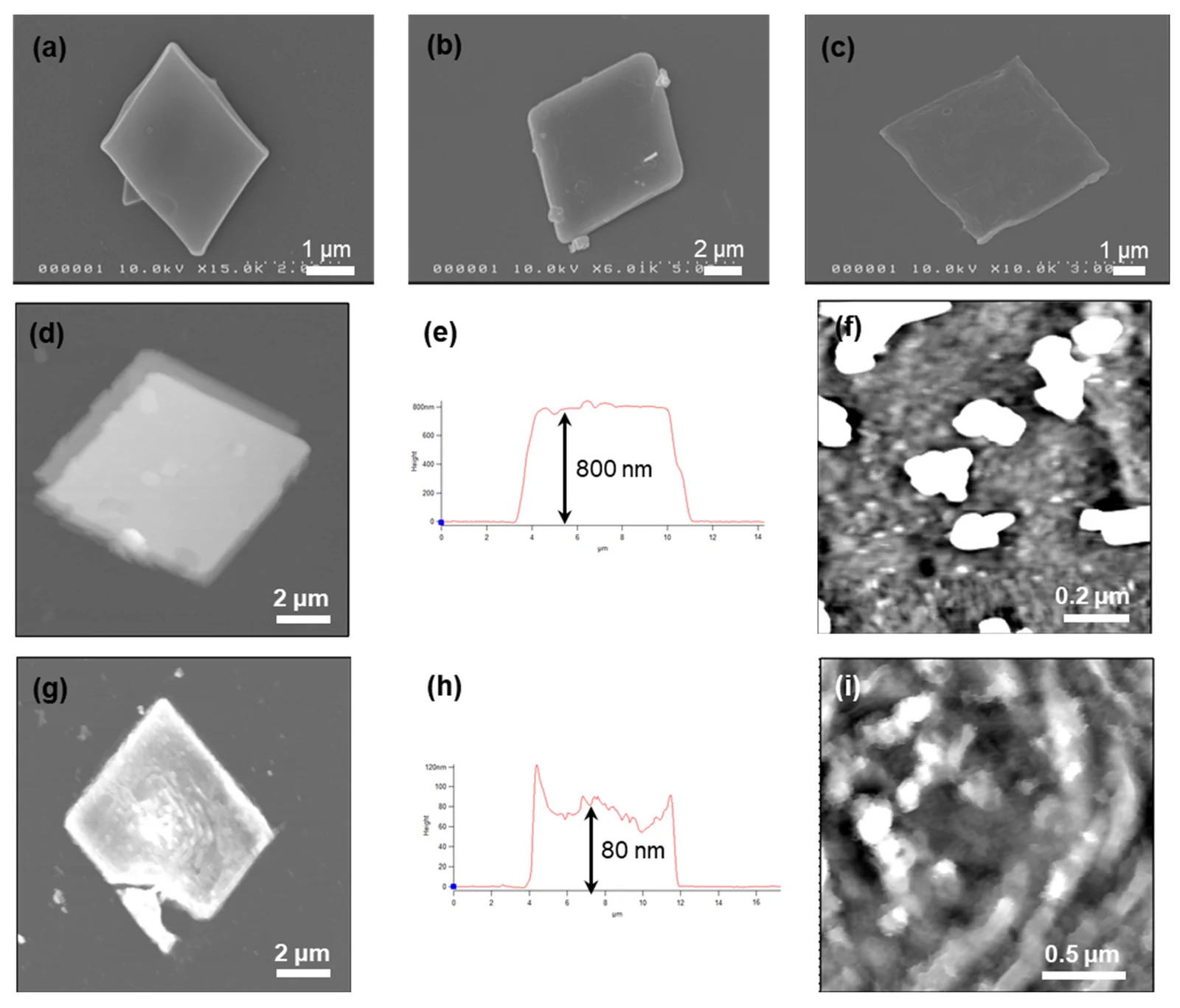
Structured conducting polymers based on the oxidant superstructure template. (**a**–**c**) Representative SEM images of plate-shaped oxidant superstructure, after the VPP of EDOT on the template, and PEDOT plate after the wash removal of oxidant, respectively. (**d**) Low magnification AFM image, (**e**) sectional height profile, and (**f**) high magnification AFM image of plate-shaped oxidant superstructure. (**g**) Low magnification AFM image, (**h**) sectional height profile, and (**i**) high magnification AFM image of the synthesized PEDOT plate after the wash removal of oxidant superstructure template.

Figure 5 shows the synthesis of structured conducting polymer, PEDOT here, using oxidant superstructures as templates. Figure 5a–c show the SEM images of an oxidant superstructure, after the VPP of EDOT monomer, and the final structured PEDOT after the wash removal of oxidant template, respectively. Further details of oxidant superstructure and the synthesized structured PEDOT could be investigated by AFM. Shown in Figure 5d–f are AFM results on oxidant superstructure surface. The plate-shaped oxidant superstructure is ~800 nm thick, with ~10 μm × 10 μm in lateral dimension. The magnified image in Figure 5f clearly shows the small nanoparticle-like features, which indicates that the superstructure is composed of nanoparticles. Figure 5g–i show the AFM data on the synthesized structured PEDOT. The synthesized PEDOT takes the shape of the template superstructure, as shown in Figure 5g. The thickness of the synthesized PEDOT was found to be ~80 nm, based on the section profile shown in Figure 5h, where the thickness of PEDOT can be easily varied by controlling synthesis conditions. Upon magnification, the surface of the synthesized PEDOT shows granular features, as shown in Figure 5i, which is the reminiscent of nanoparticles shown in Figure 5f. In addition, other conducting polymers, such as Ppy and PTTh, could also be successfully synthesized using the oxidant superstructures as templates (Figure S7).

**Figure 6:**
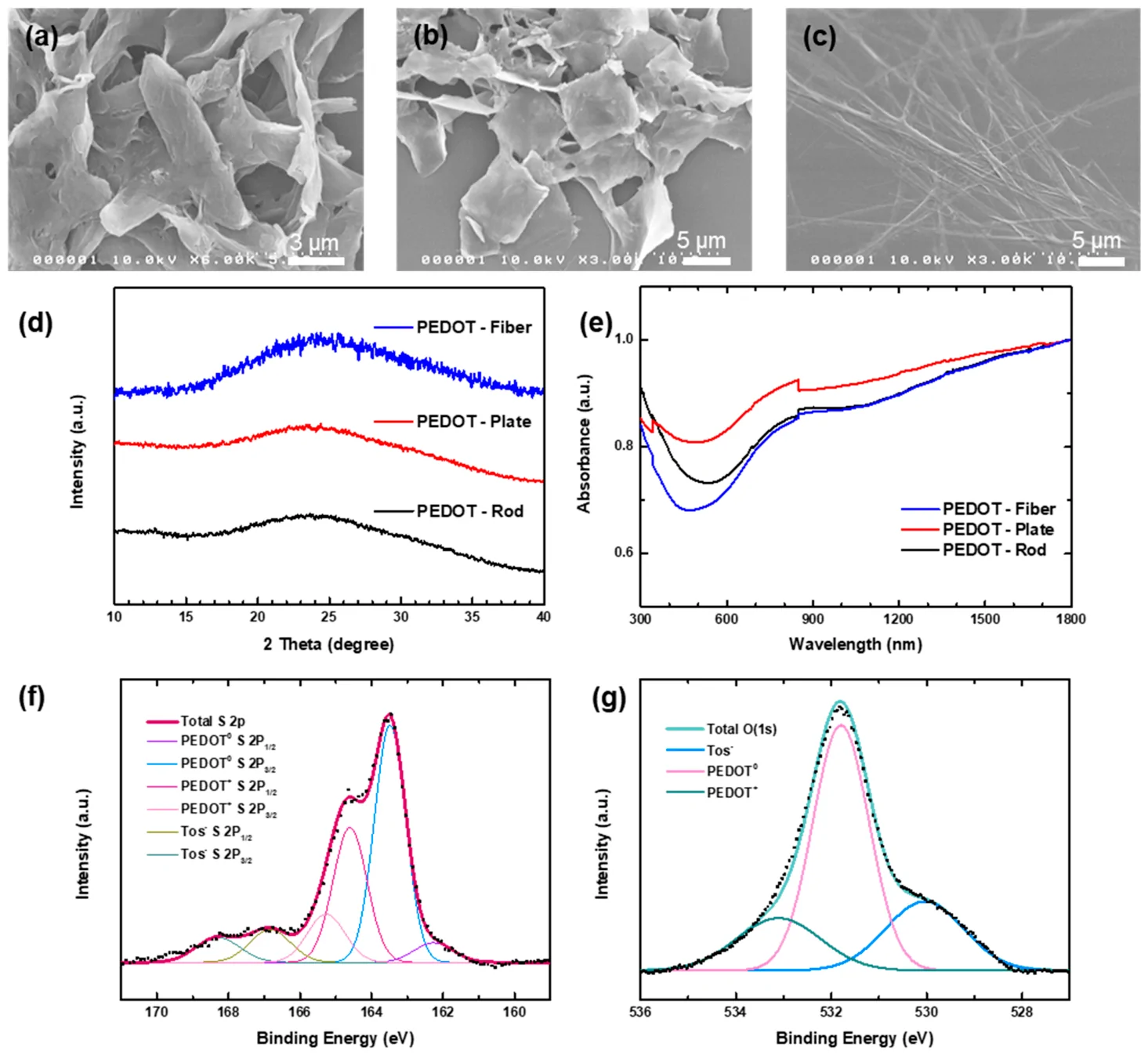
Analytic characterization of the synthesized structured PEDOT, (**a**–**c**) SEM, (**d**) XRD, (**e**) UV–Vis, and (**f**) and (**g**) XPS, respectively.

Various oxidant superstructure templates were used for the synthesis of structured PEDOT, and the results are shown in Figure 6a–c. Here, depending on the shape of the oxidant superstructure templates, the synthesized PEDOT exactly replicates the shape of the templates: rods in Figure 6a, plates in Figure 6b, and fibers in Figure 6c. Another interesting observation is the stitching among the synthesized PEDOT units, rods, plates, or fibers during the polymerization (Figure S8). This stitching can enhance the electrical conductivity by reducing the contact resistance among those units. The synthesized structured PEDOTs in different shapes, shown in Figure 6a–c, have been characterized by various analytic techniques. In the XRD spectra, shown in Figure 6d, there were no noticeable differences among the PEDOTs polymerized from oxidant superstructures having different shapes. And the main peak at 2θ~25° (d-spacing = 3.56 Å) is from the π-π stacking of the planar thiophene ring in PEDOT [37]. To investigate the doping state of the structured PEDOT, UV–Vis absorption spectra were measured and are shown in Figure 6e. The spectra show an absorption dip at ~500 nm and bumps at ~900 nm. This indicates that the polymerized PEDOT structures are primarily in the neutral state, as it is well-known that neutral PEDOT exhibits strong absorption at 500~600 nm. However, the bump observed in the UV–Vis spectra suggest that the synthesized PEDOT also contains radical cation (polaron, ~900 nm) states [38].

XPS elemental analysis was conducted by examining the oxygen O(1s) in the doped tosylate ions within PEDOT, as well as the sulfur S(2p) in the thiophene ring and tosylate ions, based on the expected atomic structure of the synthesized PEDOT. The results are shown in Figure 6f and Figure 6g, respectively. The peaks at 162.3 eV and 163.5 eV correspond to S atoms within the thiophene ring of pristine PEDOT (PEDOT_0_), while the peaks at 164.6 eV and 165.3 eV are associated with S atoms in doped PEDOT (PEDOT^+^). Additionally, the peaks at 166.8 eV and 168.3 eV represent S atoms in tosylate ions that are electrostatically bound to the PEDOT chain. The doping level of tosylate ions in the PEDOT chain can be calculated by comparing the area ratio between the thiophene and the tosylate S(2p) peaks within the binding energy range of 170–160 eV. Based on this method, the doping ratio of the PEDOT sample was estimated to be 3.5:1, which is close to the ideal doping ratio of one tosylate anion per four thiophene rings in high-conductivity PEDOT. The presence of doped tosylate ions in the PEDOT film was further confirmed by a peak at 530 eV in the O(1s) spectrum, while the peaks at 531.8 eV and 533.1 eV are associated with the C–O–C bonds in PEDOT_0_ and PEDOT^+^, respectively [39].

### 3.3. PEDOT Fibers Electrode for High-Performance Supercapacitors

**Figure 7:**
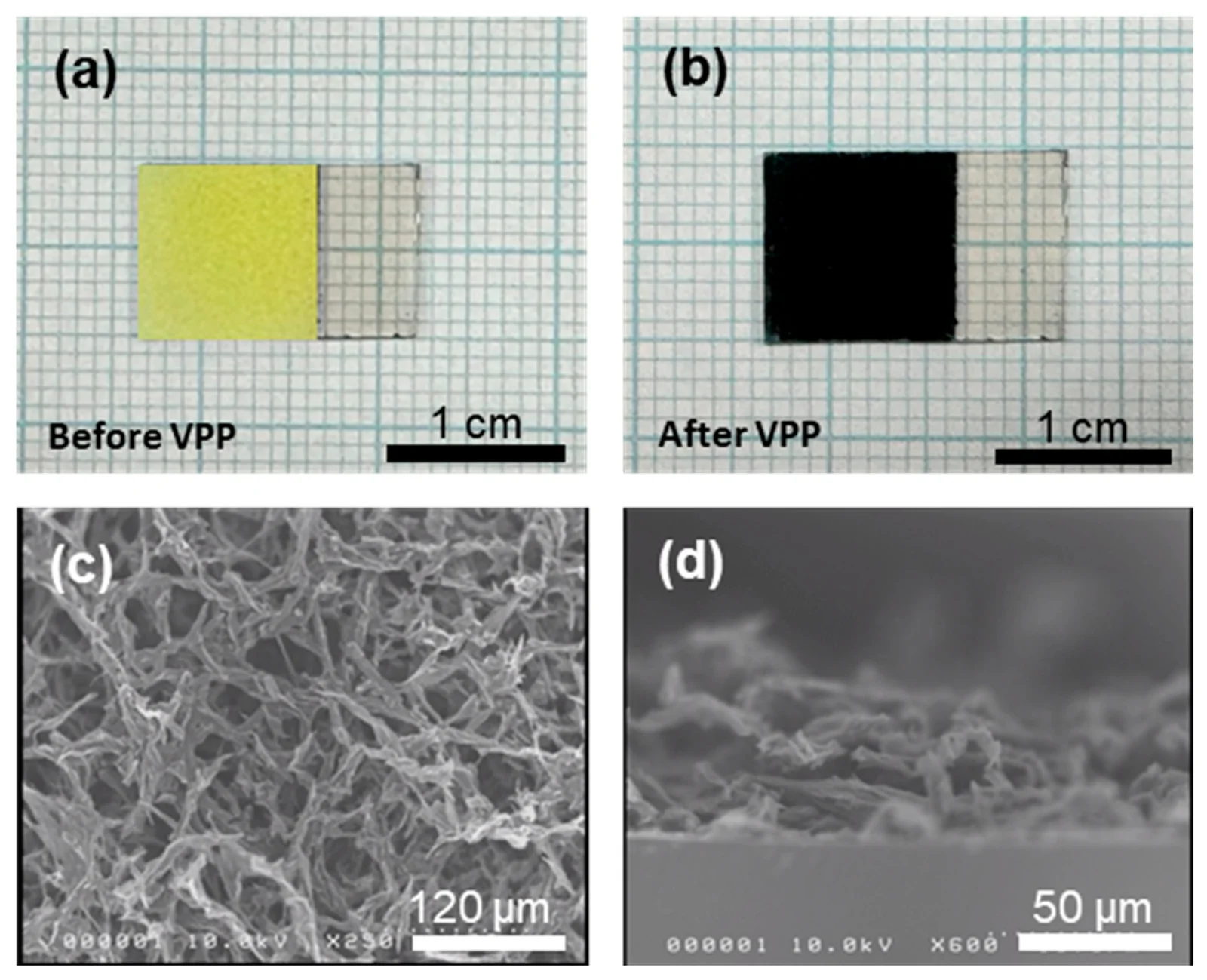
Polymerization of porous PEDOT fiber networks on ITO glass. Photo images of ITO surfaces (**a**) before and (**b**) after the polymerization. (**c**) Top view and (**d**) cross-sectional SEM images of the synthesized PEDOT fiber networks.

In supercapacitors, increasing conductivity and surface area of electrodes to facilitate the charge accumulation play a crucial role in enhancing the device performance. When fiber-shaped oxidant superstructures are used for the synthesis of a PEDOT fiber network, the fibrous structure provides the advantage of a large surface area, as well as high electrical conductivity, compared to other shapes of superstructures (Figure S9). To create this type of supercapacitor, fiber-shaped oxidant superstructures were spray-coated onto a cleaned ITO glass, followed by polymerization via the VPP process to form PEDOT fiber networks. The oxidant fiber sample area on the ITO glass was 1 cm × 1 cm, which was defined by PI tape mask (yellow region in Figure 7a). As the polymerization reaction proceeded, the color of the sample changed from yellow to deep blue (Figure 7b). When the color change was complete, it signified the completion of PEDOT synthesis. In VPP synthesis, synthesis temperature and time significantly influence the final product, resulting in variations in the shape and thickness of the synthesized PEDOT fibers (Figure S10). In the sample synthesized at 40 °C for 1 h, polymerization was insufficient, leading to very short and less-porous PEDOT fibers. In contrast, at 90 °C for 1 h, polymerization occurred too rapidly on the surface of the oxidant fibers, producing very thin PEDOT fibers. The condition that produced the highest porosity was 40 °C for 6 h, resulting in a highly porous film structure, as shown in Figure 7c,d.

**Figure 8:**
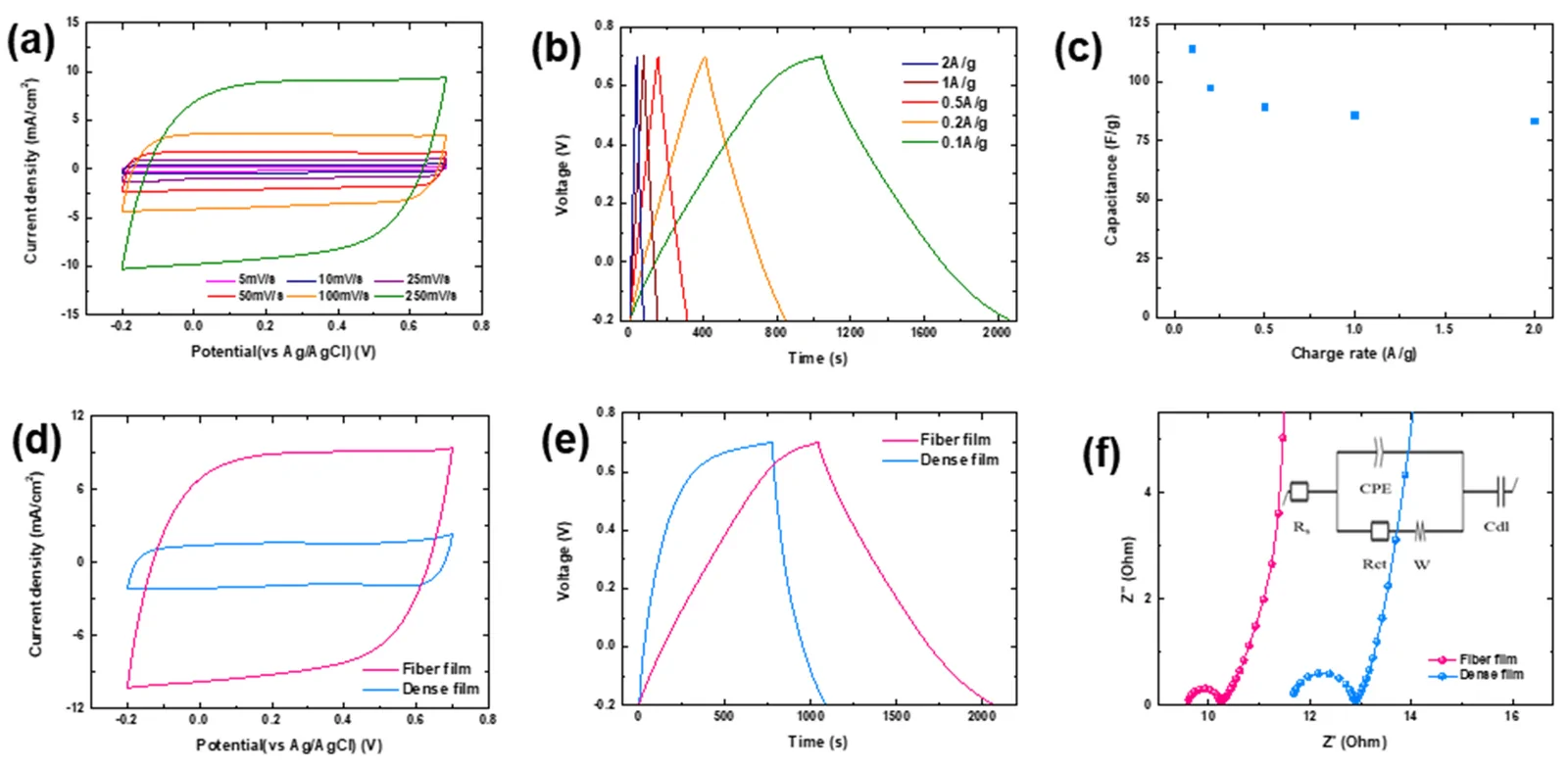
Electrical performance of porous PEDOT fibers electrode in 1.0 M KCl aqueous electrolyte. (**a**) CV curves, (**b**) galvanostatic charge–discharge (GCD) profiles, (**c**) capacitance retention. Comparison of porous PEDOT fiber film with dense PEDOT film. (**d**) CV curves, (**e**) GCD profiles and (**f**) Nyquist plots.

The performance of porous PEDOT fiber networks film as an electrode in supercapacitor was tested in 1 M KCl aqueous electrolyte using a three-electrode system (Figure S2). The CV curves of the PEDOT fiber networks electrode display a quasi-rectangular shape, as shown in Figure 8a. In the galvanostatic charge–discharge (GCD) profiles, the porous PEDOT fiber networks electrode exhibits a linear charge/discharge profile across the charge/discharge rate ranges of 0.1 A/g to 2 A/g, as shown in Figure 8b. This indicates that the PEDOT fiber networks electrode achieves charge storage via the formation of an electrical double layer in the 1 M KCl electrolyte. The specific capacitance of the supercapacitor with porous PEDOT fiber networks electrode was calculated using Equation (1) from the experimental section and shows a capacitance of 114.3 F/g at a current density of 0.1 A/g, which is ~2 times higher than that with a dense PEDOT film (54.2 F/g), suggesting its potential as a supercapacitor electrode (Table S1). Note here that the dense PEDOT film was synthesized via VPP using spun-cast dense oxidant film. The observed improvement in capacitance can be attributed to the porous (porosity of ~32%) structure of PEDOT fiber networks film compared to dense PEDOT film, allowing for greater ion deposition and the formation of more electric double layers, resulting in enhanced capacitance (Figure S11). The increased surface area due to the porous structure allows ions to access the electrolyte/electrode interface more easily, facilitating electrochemical reactions. Additionally, rate capability shows a capacitance retention of 73%, as shown in Figure 8c. Shown in Figure 8d,e is the comparison between porous and dense PEDOT electrodes, demonstrating much improved performance in porous PEDOT electrode samples.

To measure the rate capability of the two different electrodes, porous and dense PEDOT electrodes, EIS analysis was performed and is shown in Figure 8f (Figure S12 for original Nyquist plot). Nyquist plots typically consist of a semicircle at high frequencies and a vertical line at low frequencies. In the case of the porous PEDOT sample, vertical lines can be observed in the low-frequency range, indicating supercapacitor behavior. The diameter of the semicircle represents the charge transfer resistance at the interface, and the porous PEDOT sample displays a much smaller semicircle compared to the dense film sample, indicating lower contact resistance and more efficient charge distribution at the interface. Additionally, the intersection points with the Z’ axis correspond to resistance arising from the contact between the electrolyte, electrode, and substrate. It is observed that the porous PEDOT sample shows lower resistance than the dense film sample. This superior performance can be attributed to the porous structure of the PEDOT fiber networks electrode sample, which provides a larger surface area, facilitating ion movement at the electrolyte–electrode interface.

Furthermore, the impedance behavior of an electrode is determined by several elements: R_s_ (solution resistance), R_ct_ (charge transfer resistance), the Warburg impedance (resulting from ion diffusion from the electrolyte to the electrode surface), the constant phase element (representing surface roughness and porosity), and the capacitance of the electric double-layer capacitor (EDLC) formed at the electrolyte–electrode interface. These elements are represented by a modeled equivalent circuit, as shown in the inset of Figure 8f. The extracted parameters from the EIS data are tabulated in Table S2. The porous PEDOT fiber electrode exhibited a contact resistance of 9.60 Ω, lower than that for the dense PEDOT film (11.63 Ω), indicating better ion transport at the interface. Warburg impedance, representing ion diffusion, was 4.05 Ω for the porous film and 5.92 Ω for the dense film, showing that the porous structure provides shorter and more efficient ion diffusion pathways. The time constant (τ_0_), reflecting charge/discharge speed, was 0.57 s for the porous film and 1.24 s for the dense film. The smaller time constant of the porous film highlights faster charge/discharge dynamics, enabled by its larger surface area and higher porosity, which enhance ion accessibility and accelerate electrochemical reactions. Collectively, these results demonstrate that the porous structure increased surface area and makes the porous PEDOT fiber electrode highly effective for improving supercapacitor performance [34,40].

## 4. Conclusions

In summary, we successfully synthesized the structured CPs using Ouzo-induced oxidant superstructures as templates. The solvent/oxidant mixture spontaneously forms emulsion droplets in an anti-solvent medium, with no need for surfactants. However, by slightly increasing the oxidant concentration beyond the stability limit of the stable Ouzo emulsion, the internal concentration increases due to the drainage of solvent into anti-solvent, leading to nanoparticle formation. The precipitated nanoparticles form superstructures via oriented attachment. As the lattice does not have sufficient time to fully stabilize, metastable superstructures are formed first. These superstructures undergo several morphological transformations (rod, plate, and fiber) as they approach a stable state. More detailed study on the thermodynamic origin of superstructure formation and the effect of continuous stirring should be entailed later on. The iron(III) p-toluenesulfonate superstructures retain their oxidizing properties throughout these shape transformations, leaving their core functionality as an oxidant unaffected. Furthermore, we successfully synthesized CPs with diverse structural morphologies, including fibrous forms. The fibrous PEDOT films, due to their porous structure, demonstrated a significantly higher specific capacitance (114.3 F/g) compared to dense films (54.2 F/g), attributed to their increased surface area. This work is the very first report on the formation of superstructures in simple ternary systems. The demonstrated approach for the synthesis of structured CPs, using oxidant superstructures as templates, may find applications in diverse fields.

## Data Availability

The original contributions presented in this study are included in the article/Supplementary Material. Further inquiries can be directed to the corresponding author.

## Supplementary Materials

The following supporting information can be downloaded at: https://www.mdpi.com/article/10.3390/polym18182260/s1, Figure S1: Time evolution of ternary solution upon continuous stirring; Figure S2: Experimental set up for electrochemical characterization; Figure S3: SEM images for shape transformation in oxidant superstructures; Figure S4: Superstructures shape evolution for different combinations of solvent/anti-solvent combinations; Figure S5: TGA data for pristine oxidant powders and oxidant superstructures; Figure S6: FT-IR spectra over the full wavenumber range; Figure S7: SEM images of structured CPs (Ppy and PTTh); Figure S8: SEM images for stitched structured PEDOT plates; Figure S9: SEM images and electrical conductivities of PEDOT films templated from different shapes of oxidant superstructures; Figure S10: SEM images of porous PEDOT fiber films synthesized at different conditions; Figure S11: Estimation of porosity in porous PEDOT fiber film; Figure S12: EIS spectra for porous and dense PEDOT film electrodes; Table S1: Performance comparison between porous and dense PEDOT electrodes. Table S2: Extracted parameters from the equivalent circuit fitting with EIS data.

## Abbreviations

The following abbreviations are used in this manuscript:

AFM	Atomic Force Microscopy
CE	Counter Electrode
CP	Conducting Polymer
CV	Cyclic Voltammetry
EDLC	Electric Double Layer Capacitance
EDOT	Ethylenedioxythiophene
EIS	Electrochemical Impedance Spectroscopy
ESR	Equivalent Series Resistance
FT-IR	Fourier-Transform Infrared Spectroscopy
GCD	Galvanostatic Charge/Discharge
ITO	Indium Tin Oxide
PEDOT	Polyethylenedioxythiophene
Ppy	Polypyrrole
PT	Polythiophene
PTTh	Polyterthiophene
Rct	Charge Transfer Resistance
RE	Reference Electrode
Rs	Solution Resistance
SEM	Scanning Electron Microscopy
TEM	Transmission Electron Microscopy
TGA	Thermogravimetric Analysis
VPP	Vapor Phase Polymerization
WE	Working Electrode
XPS	X-ray Photoelectron Spectroscopy
XRD	X-ray Diffraction

## References

[1] Lock J.P., Im S.G., Gleason K.K. (2006). Oxidative Chemical Vapor Deposition of Electrically Conducting Poly(3,4-ethylenedioxythiophene) Films. Macromolecules.

[2] Fabretto M., Müller M., Hall C., Murphy P., Short R.D., Griesser H.J. (2010). In-situ QCM-D Analysis Reveals Four Distinct Stages During Vapour Phase Polymerisation of PEDOT Thin Films. Polymer.

[3] Howden R.M., McVay E.D., Gleason K.K. (2013). oCVD Poly(3,4-ethylenedioxythiophene) Conductivity and Lifetime Enhancement Via Acid Rinse Dopant Exchange. J. Mater. Chem. A.

[4] Li J., Zhang M., Liu J., Ma Y. (2014). Effect of Attached Peroxyacid on Liquid Phase Depositional Polymerization of EDOT over PI Film with Adsorbed Ferric Chloride. Synth. Met..

[5] Li J., Ma Y. (2016). In-Situ Synthesis of Transparent Conductive PEDOT Coating on PET Foil by Liquid Phase Depositional Polymerization of EDOT. Synth. Met..

[6] Roncali J., Blanchard P., Frère P. (2005). 3,4-Ethylenedioxythiophene (EDOT) as a Versatile Building Block for Advanced Functional Pi-Conjugated Systems. J. Mater. Chem..

[7] Mueller M., Fabretto M., Evans D., Hojati-Talemi P., Gruber C., Murphy P. (2012). Vacuum Vapour Phase Polymerization of High Conductivity PEDOT: Role of PEG-PPGPEG, the Origin of Water, and Choice of Oxidant. Polymer.

[8] Brooke R., Cottis P., Talemi P., Fabretto M., Murphy P., Evans D. (2017). Recent Advances in the Synthesis of Conducting Polymers from the Vapour Phase. Prog. Mater. Sci..

[9] Abdelhamid M.E., O’Mullane A.P., Snook G.A. (2015). Storing Energy in Plastics: A Review on Conducting Polymers & Their Role in Electrochemical Energy Storage. RSC Adv..

[10] Xia L., Wei Z., Wan M. (2010). Conducting Polymer Nanostructures and Their Application in Biosensors. J. Colloid Interface Sci..

[11] Han M.G., Foulger S.H. (2005). 1-Dimensional Structures of Poly(3,4-ethylenedioxythiophene)(PEDOT): A Chemical Route to Tubes, Rods, Thimbles, and Belts. Chem. Commun..

[12] Ghosh S., Remita H., Ramos L., Dazzi A., Deniset-Besseau A., Beaunier P., Goubard F., Auber P., Brisset F., Remita S. (2014). PEDOT Nanostructures Synthesized in Hexagonal Mesophases. New J. Chem..

[13] Zhang X., MacDiarmid A.G., Manohar S.K. (2005). Chemical Synthesis of PEDOT Nanofibers. Chem. Commun..

[14] Lee J., Cho S.-B., Dimitrov K.C., Lee Y., Khang D.-Y. (2024). Conducting Polymer Hollow Nanostructures by Surfactant-Free Ouzo Emulsion for an Exceptional EMI Shielding Performance. Eur. Polym. J..

[15] Ganachaud F., Katz J.L. (2005). Nanoparticles and Nanocapsules Created Using the Ouzo Effect: Spontaneous Emulsification as an Alternative to Ultrasonic and High-Shear Devices. ChemPhysChem.

[16] Vitale S.A., Katz J.K. (2003). Liquid Droplet Dispersions Formed by Homogeneous Liquid-Liquid Nucleation: “The Ouzo Effect”. Langmuir.

[17] Botet R. (2012). The “Ouzo Effect”, Recent Developments and Application to Therapeutic Drug Carrying. J. Phys. Conf. Ser..

[18] Cölfen H., Mann S. (2003). Higher-Order Organization by Mesoscale Self-Assembly and Transformation of Hybrid Nanostructures. Angew. Chem. Int. Ed..

[19] Cölfen H., Antonietti M. (2005). Mesocrystals: Inorganic Superstructures Made by Highly Parallel Crystallization and Controlled Alignment. Angew. Chem. Int. Ed..

[20] Niederberger M., Cölfen H. (2006). Oriented Attachment and Mesocrystals: Non-Classical Crystallization Mechanisms Based on Nanoparticle Assembly. Phys. Chem. Chem. Phys..

[21] Cölfen H., Antonietti M. (2008). Mesocrystals and Nonclassical Crystallization.

[22] Penn R.L., Banfield J.F. (1999). Morphology Development and Crystal Growth in Nanocrystalline Aggregates under Hydrothermal Conditions: Insights from Titania. Geochim. Cosmochim. Acta.

[23] Kashchiev D. (2000). Nucleation: Theory and Basic Applications.

[24] Ostwald W. (1897). Studien über die Bildung und Umwandlung fester Körper. Z. Phys. Chem..

[25] De Yoreo J., Nakouzi E., Jin B., Chun J., Mundy C. (2022). Spiers Memorial Lecture: Assembly-Based Pathways of Crystallization. Faraday Discuss..

[26] Fang J., Ding B., Gleiter H. (2011). Mesocrystals: Syntheses in Metals and Applications. Chem. Soc. Rev..

[27] Huang X.Q., Tang S.H., Mu X.L., Dai Y., Chen G.X., Zhou Z.Y., Ruan F.X., Yang Z.L., Zheng N.F. (2011). Freestanding Palladium Nanosheets with Plasmonic and Catalytic Properties. Nat. Nanotechnol..

[28] Blüh O. (1925). Einige bei der Untersuchung von Kolloiden im Wechselfeld auftretende Erscheinungen. Kolloid Z..

[29] Deng S.Z., Tjoa V., Fan H.M., Tan H.R., Sayle D.C., Olivo M., Mhaisalkar S., Wei J., Sow C.H. (2012). Reduced Graphene Oxide Conjugated Cu_2_O Nanowire Mesocrystals for High Performance NO_2_ Gas Sensor. J. Am. Chem. Soc..

[30] Teng X.W., Maksimuk S., Frommer S., Yang H. (2007). Three-Dimensional PtRu Nanostructures. Chem. Mater..

[31] Hu X.L., Gong J.M., Zhang L.Z., Yu J.C. (2008). Continuous Size Tuning of Monodisperse ZnO Colloidal Nanocrystal Clusters by a Microwave-Polyol Process and Their Application for Humidity Sensing. Adv. Mater..

[32] Uchaker E., Cao G.Z. (2014). Mesocrystals as Electrode Materials for Lithium-Ion Batteries. Nano Today.

[33] Ma M.G., Cölfen H. (2014). Mesocrystals—Applications and Potential. Curr. Opin. Colloid Interface Sci..

[34] Abdah M.A.A.M., Zubair N.A., Azman N.H.N., Sulaiman Y. (2017). Fabrication of PEDOT Coated PVA-GO Nanofiber for Supercapacitor. Mater. Chem. Phys..

[35] Xu Y., Ayala-Orozco C., Chiang P.-T., Shammai M., Wong M.S. (2020). Understanding the Role of Iron (III) Tosylate on Heavy Oil Viscosity Reduction. Fuel.

[36] Haynes J.S., Sams J.R., Thompson R.C. (1981). Synthesis and Structural Studies of Iron(II) and iron(III) Sulfonates. Can. J. Chem..

[37] Bahry T., Cui Z., Deniset-Besseau A., Gervais M., Sollogoub C., Bui T.-T., Remita S. (2018). An Alternative Radiolytic Route for Synthesizing Conducting Polymers in an Organic Solvent. New J. Chem..

[38] Massonnet N., Carella A., Jaudouin O., Rannou P., Laval G., Celle C., Simonato J.-P. (2014). Improvement of the Seebeck Coefficient of PEDOT:PSS by Chemical Reduction Combined with a Novel Method for Its Transfer Using Free-Standing Thin Films. J. Mater. Chem. C.

[39] Kim J.H., Kim H.J., Jeon J.G., Shin G., Lee J., Yun S., Kang T.J. (2021). Temperature Gradient-Driven Multilevel and Grayscale Patterning of Tosylate-Doped Poly(3,4-ethylenedioxythiophene) Films for Flexible and Functional Electronics. Adv. Mater. Technol..

[40] Choi H.S., Kim Y.H., Kim H.K., Kim K.-B. (2023). Assembly of Graphene-Wrapped ZIF-8 Microspheres and Confined Carbonization for Energy Storage Applications. J. Power Sources.

