# Supplementary material for: Structured Conducting Polymers Templated from Ouzo-Induced Oxidant Superstructures for High-Performance Supercapacitors
Source: Polymers (Basel). 2026 Sep 16;18(18):2260. doi: 10.3390/polym18182260 (PMC13611279; doi:10.3390/polym18182260)
Supplement: Supplementary file 1 [file polymers-18-02260-s001.zip › polymers-4532743-supplementary.pdf]

Supplementary Materials for

Structured conducting polymers templated from Ouzo-induced oxidant superstructures for high-performance supercapacitors

Doo-Ho Kang, Jia Lee, Dahl-Young Khang*

Department of Materials Science and Engineering, Yonsei University, Seoul 03722, Korea

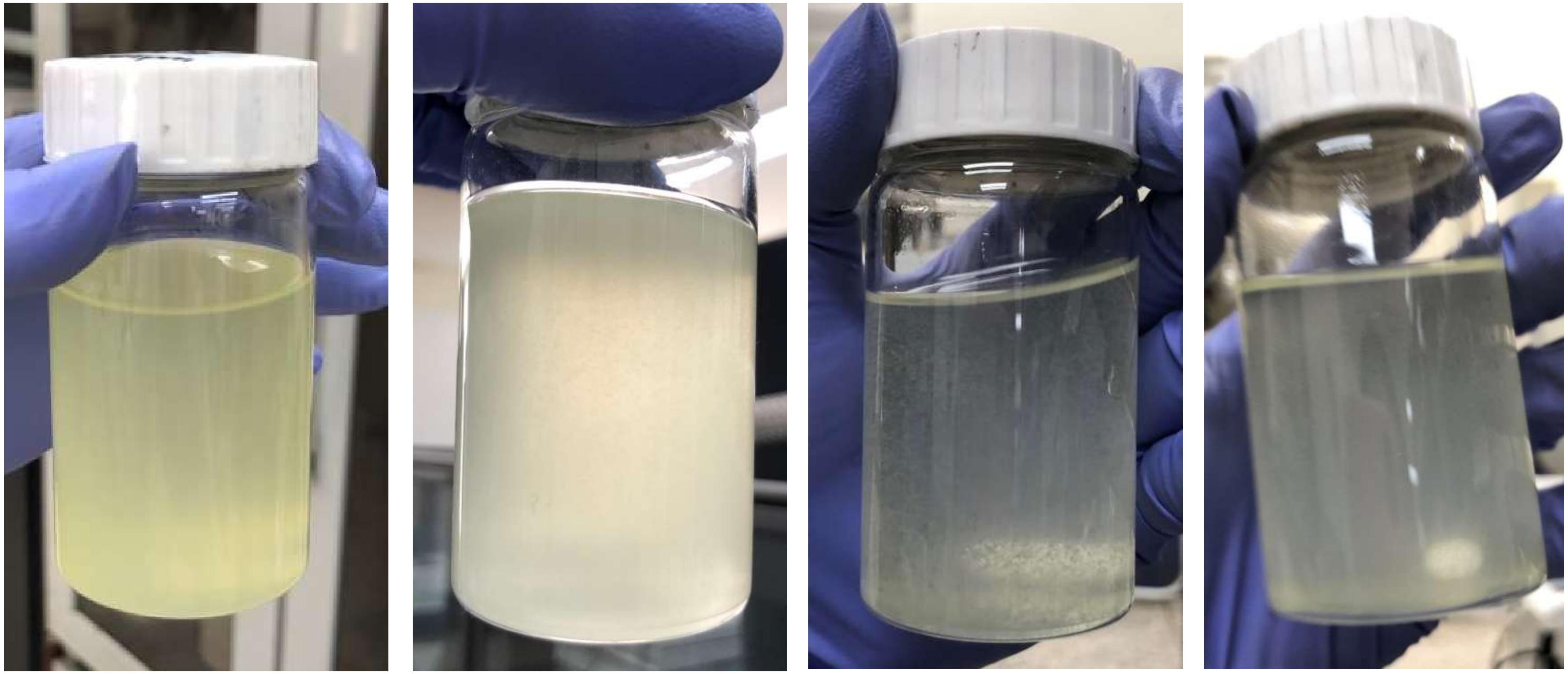

0 min 3 min 7 min 2 hrs

Fig. S1. Time evolution of ternary solution upon continuous stirring.

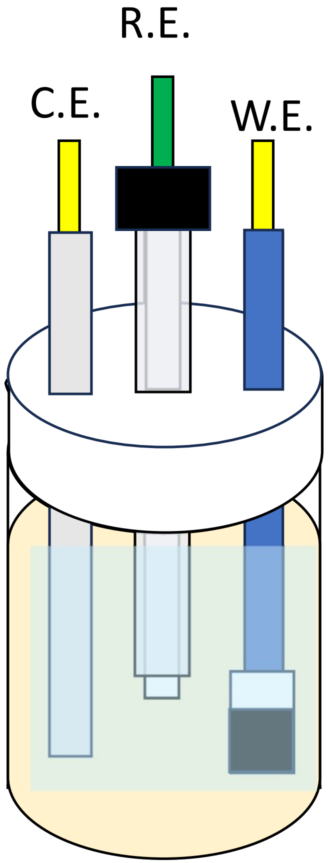

1M KCl sol.
Counter Electrode : Platinum wire
Reference Electrode : Ag/AgCl
Working Electrode : PEDOT film on ITO

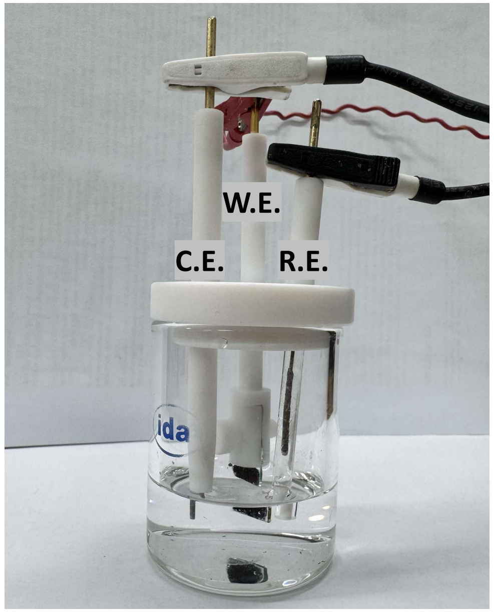

Fig. S2. Experimental setup for electrochemical characterization. (left) Schematic drawing for the 3 electrodes system, and (right) photo image of experimental 3 electrodes setup.

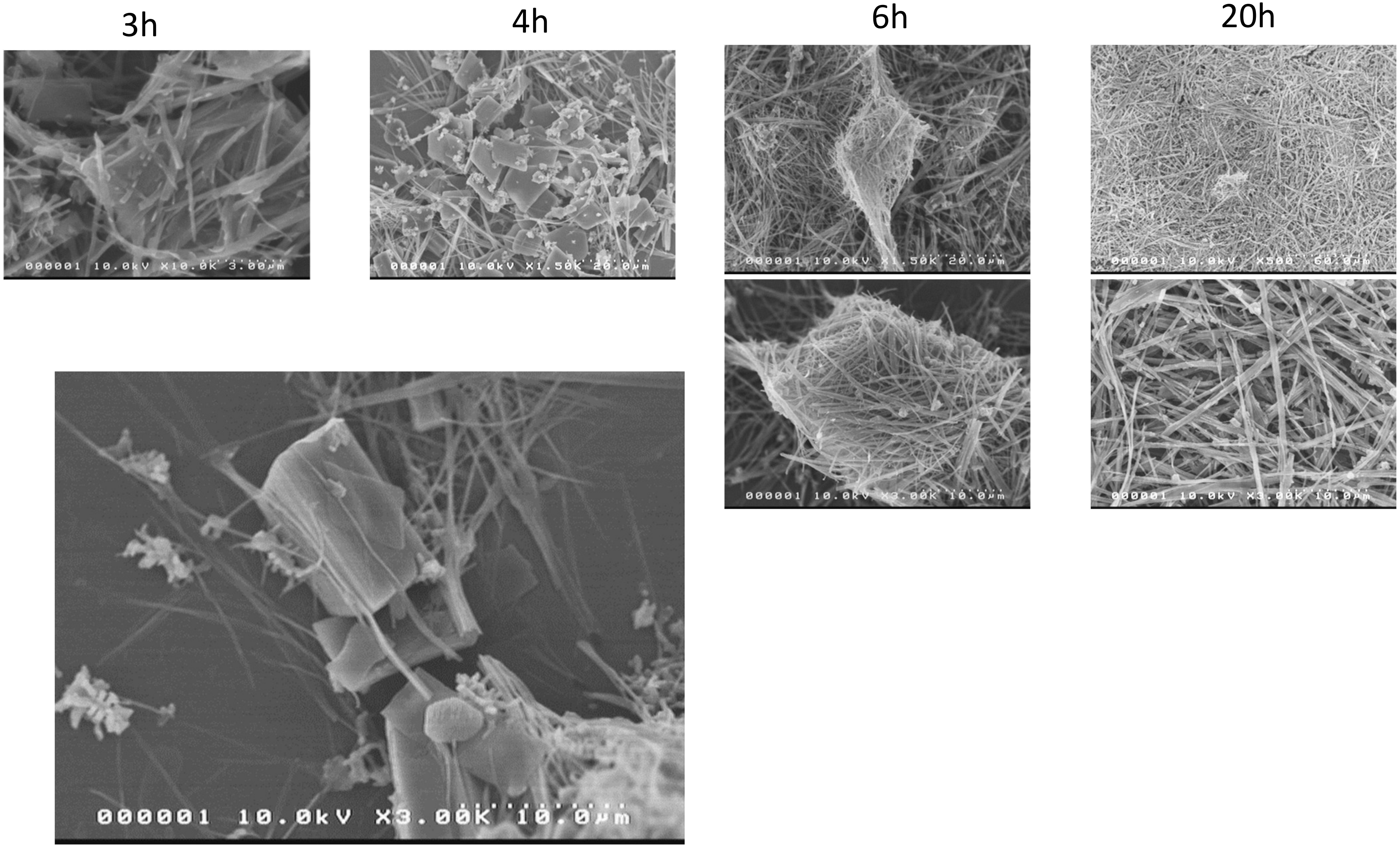

Fig. S3. Shape transformation from plates to fibers of oxidant mesocrystals. With stirring time progress, there occur fiber-shaped oxidant mesocrystals on/near the surface of plate-shaped mesocrystals.

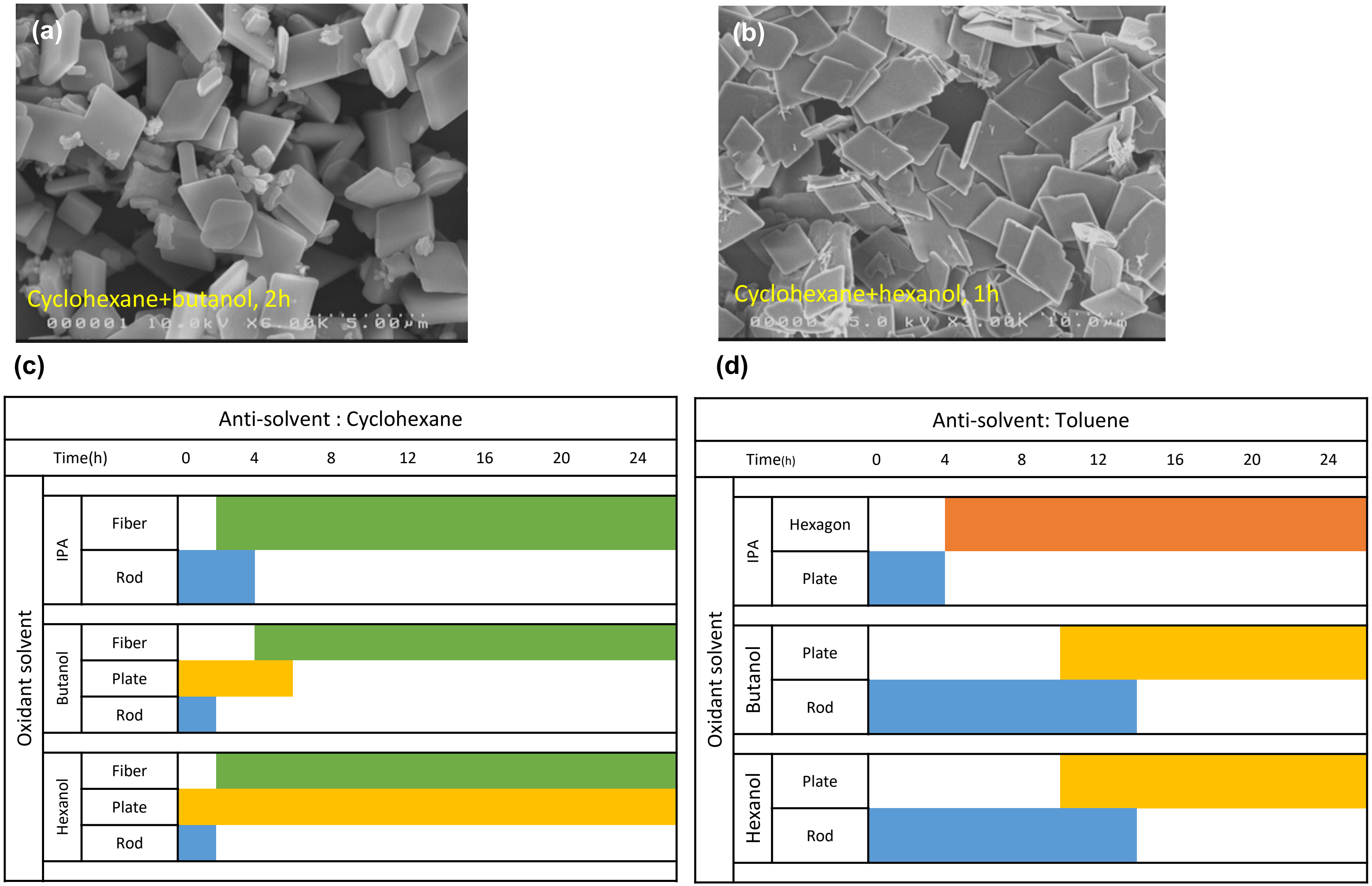

Fig. S4. Effect of solvent-antisolvent combination on the evolution dynamics and morphologies of formed oxidant mesocrystals. SEM images of plate-shaped mesocrystals formed at 1~2 hours of stirring in the cyclohexane-butanol system (a), and in the cyclohexane-hexanol system (b). (c) & (d) Shape evolution of oxidant mesocrystals depending on the solvent-antisolvent combination.

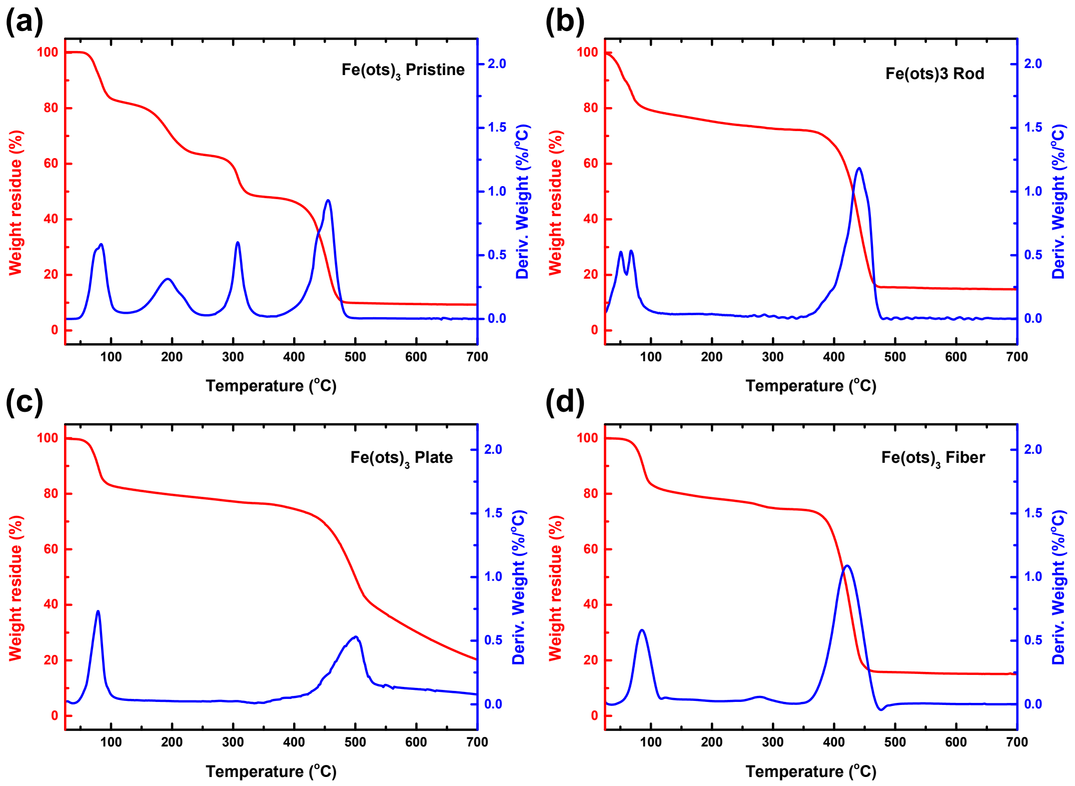

Fig. S5. TGA data for (a) pristine oxidant material, and mesocrystals of (b) rods, (c) plates, and (d) fibers, respectively.

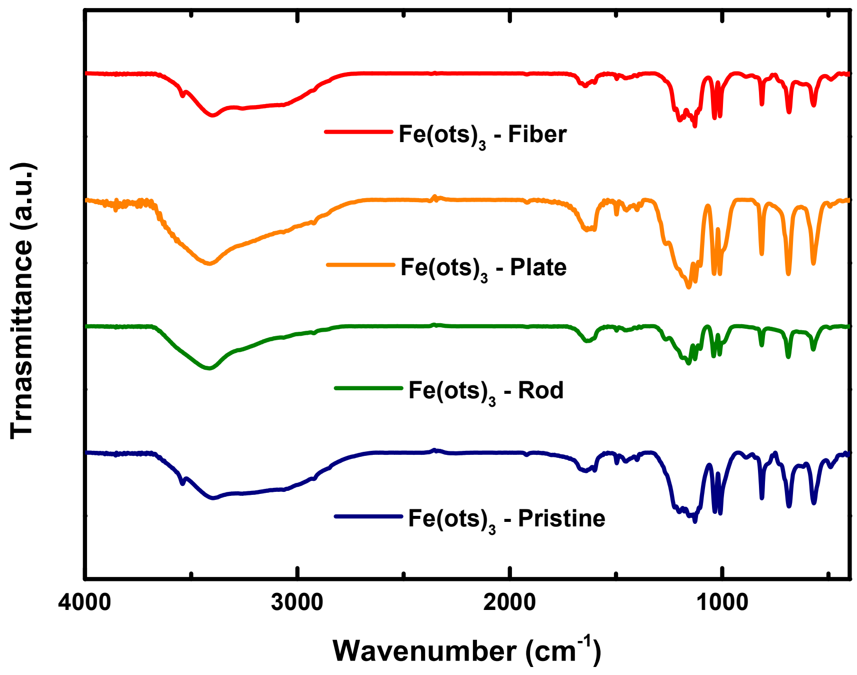

Fig. S6. FT-IR spectra of samples over the whole wavenumber range.

Polypyrrole – VPP

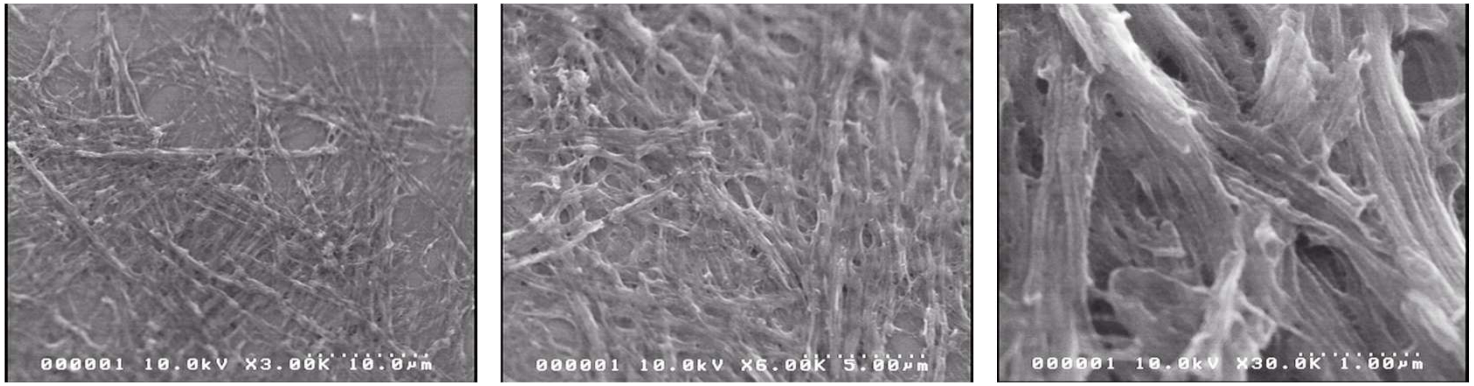

Polyterthiophene (PTTh) – VPP

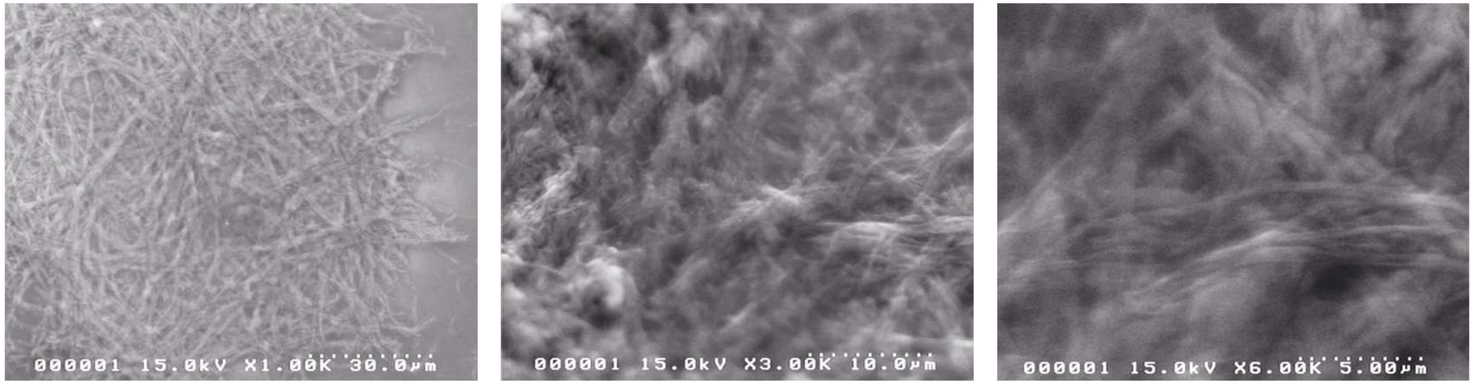

Fig. S7. SEM images conducting polymers templated from fiber-shaped oxidant mesocrystals, (top) polypyrrole, and (bottom) polyterthiophene, respectively.

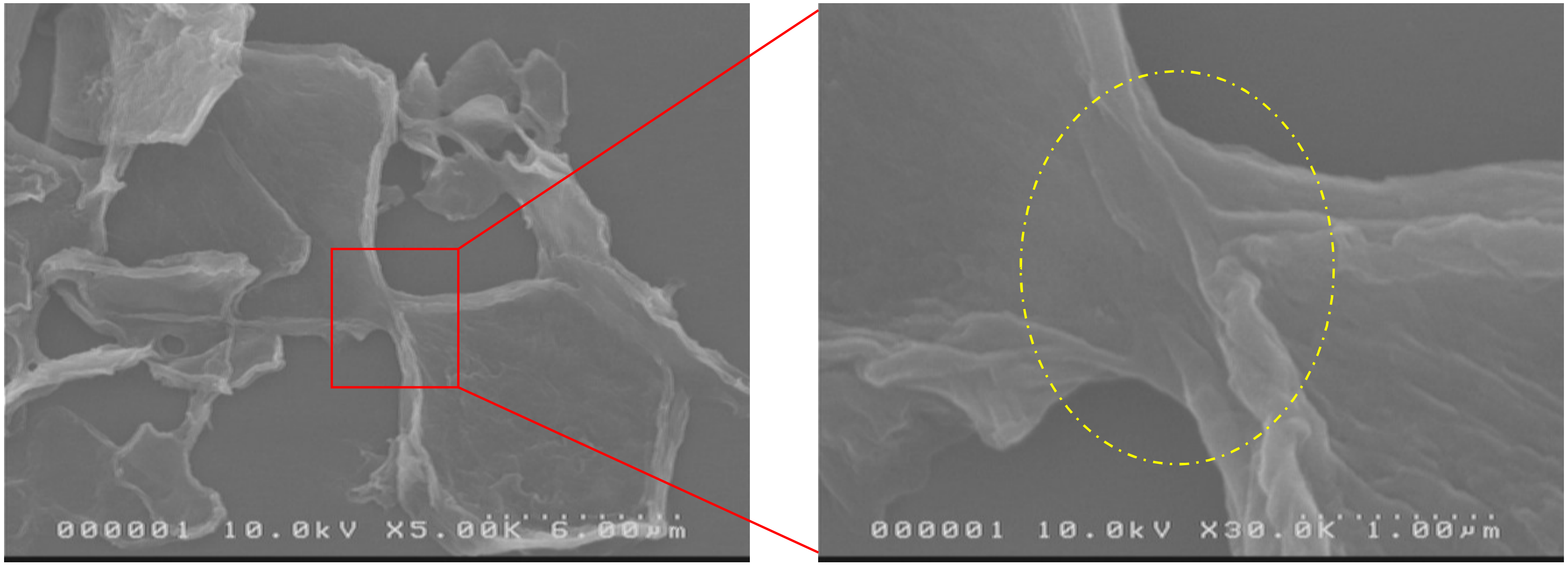

Fig. S8. SEM images of the synthesized PEDOT based on plate-shaped oxidant mesocrystals. The 2 plates are connected or stitched together during polymerization, which can enhance electrical conductivity by reducing contact resistance among the structured PEDOTs.

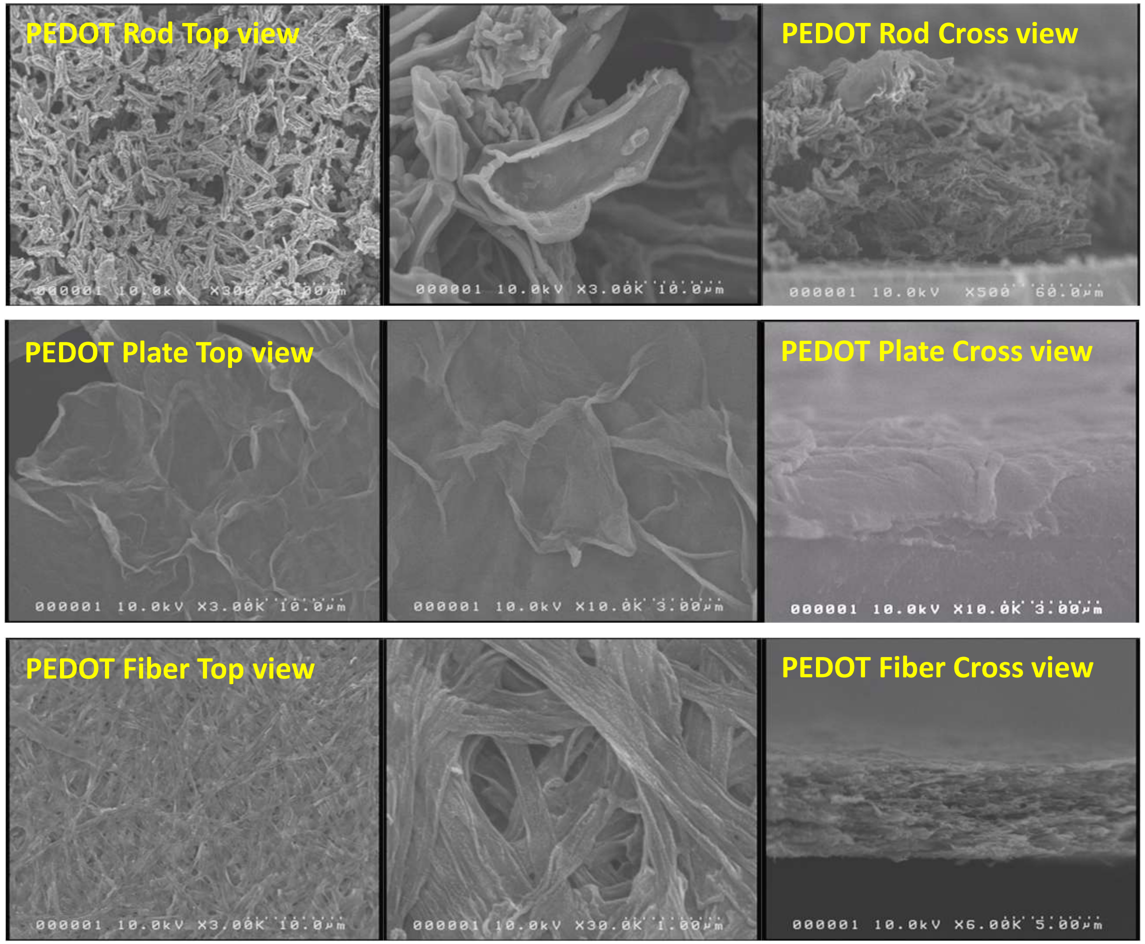

	Rod	Plate	Fiber
Thickness (μm)	81.89	1.5	5.13
Sheet resistance (Ω/□)	102.72	392.5	18.83
Conductivity (S/cm)	1.19	16.99	103.52

Fig. S9. SEM images of rods (top), plates (middle), and fibers (bottom) of PEDOT, which were polymerized based on each shape of oxidant mesocrystals as templates. Electrical conductivities of different-shaped PEDOT films via VPP on various oxidant mesocrystal templates.

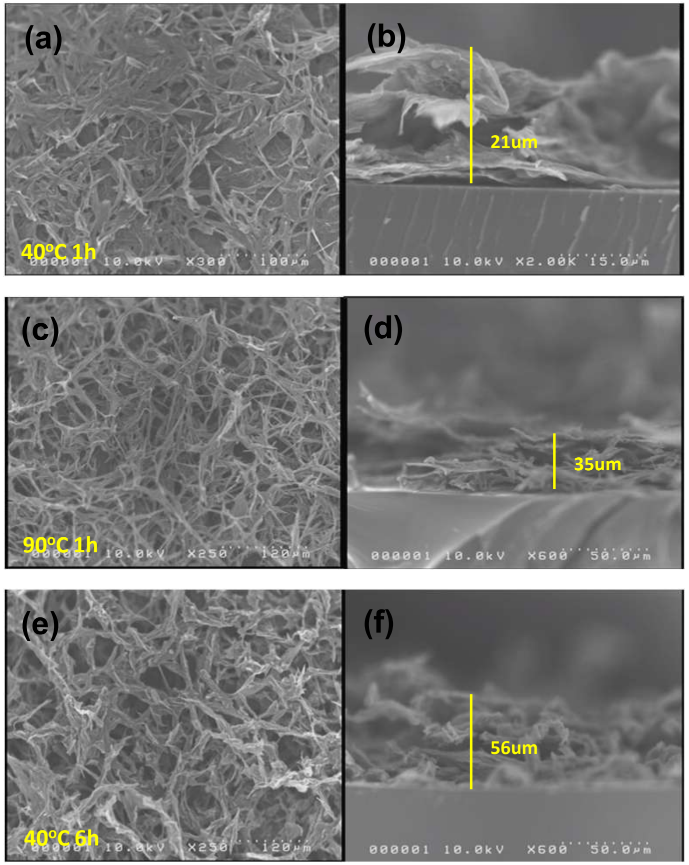

Fig. S10. SEM images (top and cross-sectional views) of porous PEDOT fiber films on ITO glass, synthesized under different conditions : (a),(b) 40°C 1h, (c),(d) 90°C 1h and (e),(f) 40°C 6h.

Porosity Calculation

To calculate the porosity , the following formula can be used:

$$\text{Porosity} = 1 - \left(\frac{\text{Porous film density}}{\text{Dense film density}}\right)$$

$$= 1 - \left(\frac{\frac{\text{Porous film Mass}}{\text{Porous film volume}}}{\frac{\text{Dense film Mass}}{\text{Dense film volume}}}\right)$$

Where:

•**Dense film :** 200μg of material is spread in a film form with a thickness of 19μm on a 1cm x 1cm substrate as a porous structure

•**Porous film :** 400μg of material is spread in a film form with a thickness of 56μm on a 1cm x 1cm substrate as a porous structure

$$\text{Porosity} = 1 - \left(\frac{0.0714}{\mathbf{0.1}053}\right) = 1 - 0.678 = 0.322$$

Thus, the porosity of the sample is approximately **32.2%**.

Fig. S11. Calculation for the porosity of PEDOT fiber films used in supercapacitors.

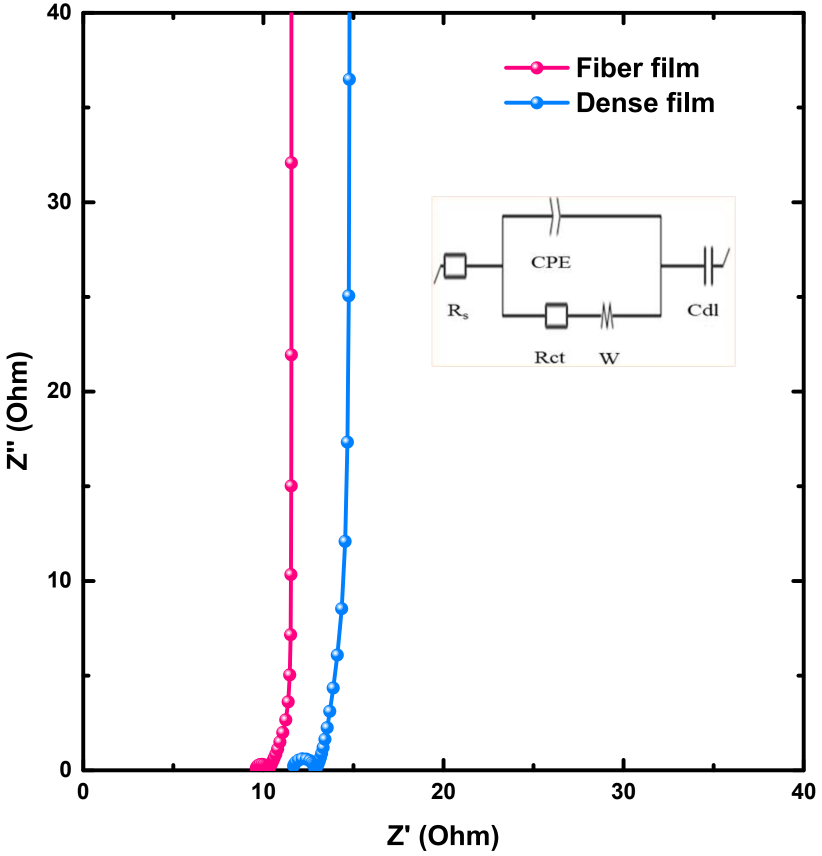

Fig. S12. EIS Nyquist plot with equi-distant real and imaginary impedance axes.

Table S1. Performance comparison between porous and dense PEDOT electrodes.

Sample type	Mass loading (μg/cm^2)	Thickness (μm)	C_s at 0.1 A/g (F/g)	Areal capacitance (mF/cm^2)	Volumetric capacitance (F/cm^3)
porous	400	56	114.3	45.7	8.2
dense	200	19	54.2	10.8	5.7

Table S2. Extracted parameters from the equivalent circuit fitting with EIS data.

	R_{ct}	ESR	CPE	W	τ_0	C_{dl}	χ^2
Porous PEDOT fiber	0.618 Ω	9.59 Ω	7.72e-6 F	4.05Ω	0.57s	0.044F	0.0041
Dense PEDOT film	1.204Ω	11.63 Ω	3.43e-6 F	5.92Ω	1.24s	0.022F	0.0059
